# Post-traumatic stress and future substance use outcomes: leveraging antecedent factors to stratify risk

**DOI:** 10.3389/fpsyt.2024.1249382

**Published:** 2024-03-08

**Authors:** Henri M. Garrison-Desany, Jacquelyn L. Meyers, Sarah D. Linnstaedt, Stacey L. House, Francesca L. Beaudoin, Xinming An, Donglin Zeng, Thomas C. Neylan, Gari D. Clifford, Tanja Jovanovic, Laura T. Germine, Kenneth A. Bollen, Scott L. Rauch, John P. Haran, Alan B. Storrow, Christopher Lewandowski, Paul I. Musey, Phyllis L. Hendry, Sophia Sheikh, Christopher W. Jones, Brittany E. Punches, Robert A. Swor, Nina T. Gentile, Lauren A. Hudak, Jose L. Pascual, Mark J. Seamon, Erica Harris, Claire Pearson, David A. Peak, Robert M. Domeier, Niels K. Rathlev, Brian J. O’Neil, Paulina Sergot, Leon D. Sanchez, Steven E. Bruce, Jutta Joormann, Steven E. Harte, Samuel A. McLean, Karestan C. Koenen, Christy A. Denckla

**Affiliations:** ^1^ Department of Social and Behavioral Sciences, Harvard T.H. Chan School of Public Health, Boston, MA, United States; ^2^ Department of Psychiatry and Behavioral Sciences, State University of New York Downstate Medical Center, New York City, NY, United States; ^3^ Department of Anesthesiology, Institute for Trauma Recovery, University of North Carolina at Chapel Hill, Chapel Hill, NC, United States; ^4^ Department of Emergency Medicine, Washington University School of Medicine, St. Louis, MO, United States; ^5^ Department of Epidemiology, Brown University, Providence, RI, United States; ^6^ Department of Emergency Medicine, Brown University, Providence, RI, United States; ^7^ Department of Biostatistics, Gillings School of Global Public Health, University of North Carolina, Chapel Hill, NC, United States; ^8^ Departments of Psychiatry and Neurology, University of California San Francisco, San Francisco, CA, United States; ^9^ Department of Biomedical Informatics, Emory University School of Medicine, Atlanta, GA, United States; ^10^ Department of Biomedical Engineering, Georgia Institute of Technology and Emory University, Atlanta, GA, United States; ^11^ Department of Psychiatry and Behavioral Neurosciences, Wayne State University, Detroit, MI, United States; ^12^ Institute for Technology in Psychiatry, McLean Hospital, Belmont, MA, United States; ^13^ The Many Brains Project, Belmont, MA, United States; ^14^ Department of Psychiatry, Harvard Medical School, Boston, MA, United States; ^15^ Department of Psychology and Neuroscience, University of North Carolina at Chapel Hill, Chapel Hill, NC, United States; ^16^ Department of Sociology, University of North Carolina at Chapel Hill, Chapel Hill, NC, United States; ^17^ Department of Psychiatry, McLean Hospital, Belmont, MA, United States; ^18^ Department of Emergency Medicine, University of Massachusetts Chan Medical School, Worcester, MA, United States; ^19^ Department of Emergency Medicine, Vanderbilt University Medical Center, Nashville, TN, United States; ^20^ Department of Emergency Medicine, Henry Ford Health System, Detroit, MI, United States; ^21^ Department of Emergency Medicine, Indiana University School of Medicine, Indianapolis, IN, United States; ^22^ Department of Emergency Medicine, University of Florida College of Medicine -Jacksonville, Jacksonville, FL, United States; ^23^ Department of Emergency Medicine, Cooper Medical School of Rowan University, Camden, NJ, United States; ^24^ Department of Emergency Medicine, Ohio State University College of Medicine, Columbus, OH, United States; ^25^ Department of Emergency Medicine, Oakland University William Beaumont School of Medicine, Rochester, MI, United States; ^26^ Department of Emergency Medicine, Lewis Katz School of Medicine, Temple University, Philadelphia, PA, United States; ^27^ Department of Emergency Medicine, Emory University School of Medicine, Atlanta, GA, United States; ^28^ Department of Surgery, University of Pennsylvania, Philadelphia, PA, United States; ^29^ Department of Neurosurgery, University of Pennsylvania, Philadelphia, PA, United States; ^30^ Perelman School of Medicine, University of Pennsylvania, Philadelphia, PA, United States; ^31^ Department of Surgery, Division of Traumatology, Surgical Critical Care and Emergency Surgery, University of Pennsylvania, Philadelphia, PA, United States; ^32^ Department of Emergency Medicine, Einstein Medical Center, Philadelphia, PA, United States; ^33^ Department of Emergency Medicine, Wayne State University, Ascension St. John Hospital, Detroit, MI, United States; ^34^ Department of Emergency Medicine, Massachusetts General Hospital, Boston, MA, United States; ^35^ Department of Emergency Medicine, Trinity Health-Ann Arbor, Ypsilanti, MI, United States; ^36^ Department of Emergency Medicine, University of Massachusetts Medical School-Baystate, Springfield, MA, United States; ^37^ Department of Emergency Medicine, Wayne State University, Detroit Receiving Hospital, Detroit, MI, United States; ^38^ Department of Emergency Medicine, McGovern Medical School at The University of Texas Health Science Center, Houston, TX, United States; ^39^ Department of Emergency Medicine, Harvard Medical School, Boston, MA, United States; ^40^ Department of Psychological Sciences, University of Missouri - St. Louis, St. Louis, MO, United States; ^41^ Department of Psychology, Yale University, New Haven, CT, United States; ^42^ Department of Anesthesiology, University of Michigan Medical School, Ann Arbor, MI, United States; ^43^ Department of Internal Medicine-Rheumatology, University of Michigan Medical School, Ann Arbor, MI, United States; ^44^ Department of Emergency Medicine, University of North Carolina at Chapel Hill, Chapel Hill, NC, United States; ^45^ Department of Psychiatry, Institute for Trauma Recovery, University of North Carolina at Chapel Hill, Chapel Hill, NC, United States; ^46^ Department of Epidemiology, Harvard T.H. Chan School of Public Health, Harvard University, Boston, MA, United States

**Keywords:** post-traumatic stress disorder, substance use, causal forest, effect modification, socioenvironmental factors, tobacco, alcohol, cannabis

## Abstract

**Background:**

Post-traumatic stress disorder (PTSD) and substance use (tobacco, alcohol, and cannabis) are highly comorbid. Many factors affect this relationship, including sociodemographic and psychosocial characteristics, other prior traumas, and physical health. However, few prior studies have investigated this prospectively, examining new substance use and the extent to which a wide range of factors may modify the relationship to PTSD.

**Methods:**

The Advancing Understanding of RecOvery afteR traumA (AURORA) study is a prospective cohort of adults presenting at emergency departments (N = 2,943). Participants self-reported PTSD symptoms and the frequency and quantity of tobacco, alcohol, and cannabis use at six total timepoints. We assessed the associations of PTSD and future substance use, lagged by one timepoint, using the Poisson generalized estimating equations. We also stratified by incident and prevalent substance use and generated causal forests to identify the most important effect modifiers of this relationship out of 128 potential variables.

**Results:**

At baseline, 37.3% (N = 1,099) of participants reported likely PTSD. PTSD was associated with tobacco frequency (incidence rate ratio (IRR): 1.003, 95% CI: 1.00, 1.01, p = 0.02) and quantity (IRR: 1.01, 95% CI: 1.001, 1.01, p = 0.01), and alcohol frequency (IRR: 1.002, 95% CI: 1.00, 1.004, p = 0.03) and quantity (IRR: 1.003, 95% CI: 1.001, 1.01, p = 0.001), but not with cannabis use. There were slight differences in incident compared to prevalent tobacco frequency and quantity of use; prevalent tobacco frequency and quantity were associated with PTSD symptoms, while incident tobacco frequency and quantity were not. Using causal forests, lifetime worst use of cigarettes, overall self-rated physical health, and prior childhood trauma were major moderators of the relationship between PTSD symptoms and the three substances investigated.

**Conclusion:**

PTSD symptoms were highly associated with tobacco and alcohol use, while the association with prospective cannabis use is not clear. Findings suggest that understanding the different risk stratification that occurs can aid in tailoring interventions to populations at greatest risk to best mitigate the comorbidity between PTSD symptoms and future substance use outcomes. We demonstrate that this is particularly salient for tobacco use and, to some extent, alcohol use, while cannabis is less likely to be impacted by PTSD symptoms across the strata.

## 1 Introduction

Traumatic events affect nearly 70% of people in a given year (1). It is estimated that 4.6% of trauma-exposed people report subthreshold post-traumatic stress (PTS) symptoms (2), and 5.6% will experience post-traumatic stress disorder (PTSD) (3). PTS is associated with numerous behavioral health outcomes, including substance use behaviors. Substance use has a higher prevalence rate among those with traumatic event exposures compared to those without (4), but the direction of effects remains unclear. Two primary hypotheses have emerged: individuals who are exposed to a traumatic event may use substances as a way to cope with the stress (5, 6), or individuals who use substances already are more likely to develop PTSD symptoms later (7), potentially through an increased risk of future traumatic events and re-traumatization, such as motor vehicle collision due to intoxication (8). To disentangle this relationship, it is important to use prospective cohort data to follow trends through time and identify differences between incident patterns, defined as new cases of substance use only when previously there was no use, and prevalent patterns, defined as both new and existing behaviors.

The relationship between substance use and PTSD, however, can be influenced by a wide range of factors. Not only is there evidence that sociodemographic factors, such as gender and age, modify this relationship (9–11), but measures of personal resiliency (12) and social support (13) have also been shown to modify this risk. However, it is not clear which of these factors, or others that have previously not been considered, are most important for moderating the risk of substance use with increased PTS symptoms. Understanding this risk stratification is critical for addressing substance use among communities exposed to traumatic events and will aid in developing treatment protocols and guide policy decisions at the population level by helping direct resources to interventions focused on groups at higher risk of substance use after traumatic events.

Prior studies, however, have also lacked prospectively collected data following a traumatic event to stratify this risk longitudinally to fully address this question. Rather, many observational studies examining the comorbidity over time have i) sampled from populations in substance use disorder or PTSD treatment programs (14, 15) and may demonstrate the most severe forms of exposure and outcome; ii) been focused primarily in veteran cohorts, which may not be generalizable to a civilian population (16–20); or iii) been analyzed cross-sectional data when PTSD and substance use were ascertained and therefore lacked a temporal relationship (21–23).

Therefore, to address these gaps in knowledge, we used a national sample of individuals presenting within 72 hours of a traumatic event to 29 emergency departments throughout the country with prospective follow-up, which allowed us to investigate incident and prevalent associations after trauma between substance use and PTSD symptoms. We hypothesized that not only sociodemographic factors but also variables related to psychological resiliency, overall health and sleep, and others may statistically modify the relationship between PTSD and substance use. We hypothesized that these effects will be the most pronounced for new cases of substance use, compared to ongoing substance use.

## 2 Methods

### 2.1 Study sample

The AURORA cohort has been previously described in detail (24). In brief, AURORA is a prospective cohort comprised of nearly 3,000 individuals who present at one of 29 participating emergency departments (EDs) within 72 hours of experiencing a traumatic event. Adults aged 18 to 75 years were excluded if they were administered general anesthesia at the time; experienced long bone fractures, significant hemorrhage, or solid organ injury; or were not alert or oriented at the time of enrollment. Individuals were observed for 1 year, with follow-up at week 2, week 8, month 3, month 6, and month 12 following enrollment in the ED. AURORA included a self-reported questionnaire and biospecimen collection of blood and saliva. Participants also consented to have their medical records included in the data collection.

### 2.2 Measures

Exposure was defined as the raw score of the self-reported PTSD checklist for DSM-5 (PCL-5) (25), reported for the 30 days prior to the event at enrollment ED visit, in the 30 days prior to week 2, week 8, month 3, month 6, and month 12. The PCL-5 was primarily used as a raw score of 0 to 80; however, we also investigated the association of baseline PCL-5 symptoms above the validated threshold of 33 (26). Using the continuous score, we maintained granularity in describing the effects across a spectrum, including subclinical presentations that still affect daily life.

Substance use was defined across three primary outcomes: tobacco, alcohol, and cannabis. These were ascertained as a self-reported count of the frequency of use and as a count of quantity of use in the last 30 days, assessed at all six timepoints using the PhenX Toolkit for evaluation (27). The quantity of cannabis was not collected as part of the PhenX Substance Abuse and Addiction Core Tier 1 questions used in the parent study protocol; therefore, our analysis focused on frequency for comparability across substances.

The following potential confounders were examined: participant age, participant gender identity (defined as cisgender men, cisgender women, and transgender/non-binary people), participant marital status (defined as never married, married, separated/divorced, and widowed/other), participant education (defined as not attending high school, attending only high school, attending college, and attending graduate school), and household income status (according to the following categories: ≤$35,000/year, >$35,000 and ≤$75,000/year, >$75,000/year, and “did not know”). Participant race/ethnicity was defined as Hispanic, non-Hispanic White, non-Hispanic Black/African-American, non-Hispanic Asian, and Native American/American Indian; other races not listed were grouped as a single category.

We investigated 128 variables in total as potential modifiers of the relationship between PTSD and substance use (**Supplementary Table 3**). The main domains of interest that the study questionnaire covered included the Connor–Davidson Resilience Scale 10 (CD-RISC-10) (28), the Five Facet Mindfulness Questionnaire (FFMQ) as a three-item numeric scale (29), the PROMIS item bank for depression and anxiety (30, 31), the Short Form Survey (SF)-12 to measure overall patient wellbeing (32), the Childhood Trauma Questionnaire Short-Form (CTSQF) (33, 34), the emotional support section of the Perceived Social Support scale (35), the Pittsburgh Sleep Quality Index (36), the Area Deprivation Index 2019 (37) linked via census tract based on a self-reported address at baseline and analyzed as national percentiles reflecting neighborhood deprivation, and the previously reported sociodemographic factors.

### 2.3 Statistical methods

Descriptive statistics were generated for exposure, outcomes, and covariates of interest. Bivariate associations between sociodemographic variables and likely PTSD diagnosis at baseline were tested using an appropriate Student’s t-test for continuous variables and a chi-squared test for categorical variables.

A sensitivity analysis, reported in the **Supplementary Material**, examined additional substances in the AURORA cohort, including opiates, cocaine, hallucinogens, stimulant drugs, barbiturates, and sedatives, in addition to tobacco, alcohol, and cannabis, using principal component analysis (**Supplementary Figure 1**). Results indicated a primary factor indexing tobacco, alcohol, and cannabis and a secondary factor indexing all other substances (opiates, cocaine, hallucinogens, stimulant drugs, barbiturates, and sedatives). These additional substances had a low frequency of use overall, with small sample sizes (N’s ranging from 27 to 105). We also assessed correlations between pairwise combinations of substances using a correlogram and found similarly that tobacco, alcohol, and cannabis use frequencies were correlated with one another but not correlated with any other individual substances (**Supplementary Figure 2**). Therefore, we proceeded with the analysis focused on the factor 1 substances.

We used generalized estimating equations (GEEs) to account for the longitudinal nature of our data, which included multiple observations for each participant. We determined autocorrelation as exchangeable via graphs and estimated the autocorrelation coefficient. We estimated GEE models, controlling for major sociodemographic covariates (gender, age, race/ethnicity, income, and marital status) and two versions of a cross-lagged model.

The first cross-lagged model used PTS symptoms from timepoints week 2, week 8, month 3, and month 6 to predict substance use at timepoints week 8, month 3, month 6, and month 12. Antecedent risk factors were defined at baseline ED visit and week 2. Therefore, at week 2, there was an overlap in risk factor responses and PTS symptom ascertainment, and the model used four timepoints. This model used the maximum number of timepoints while still maintaining the correct temporal order, although there was an overlap between antecedent factors and PTS symptoms in week 2.

The second model used antecedent risk factors at ED baseline and week 2. We used PTS symptoms at week 8, month 3, and month 6. We predicted substance use at month 3, month 6, and month 12. This model had no overlap in PTS symptoms and earlier risk factors and used three timepoints as a more stringent temporal order. We fit both models, given that the former is closer temporally to the index trauma event but has overlapping ascertainment of the risk stratification variables and the exposure, while the latter model may show less association due to increased temporal distance from the index event but maintains greater separation between antecedent and concurrent timepoints. We used the Poisson GEEs with robust standard errors given that counts of frequency and quantity of use were our primary outcomes of interest. We considered using the negative binomial GEE model as well, given that this relaxes dispersion assumptions; however, it could not converge for the data, including when amending the optimizer and attempting different model specifications.

We also compared incident and prevalent substance use and associations with PTS symptoms. “Incident” substance use was defined as having reported no past-month substance use at baseline but with later use at one or more subsequent timepoints. Only past 30-day use was considered due to the very low number of participants reporting no substance use in their lifetime. “Prevalent” substance use was defined as endorsing use at baseline and at least one subsequent timepoint.

### 2.4 Causal forest models

We conducted an honest causal forest analysis to identify the most important factors that stratify the risk of increased substance use due to PTSD symptoms. Causal forests are similar to random forests in that they aggregate a number of causal trees that iteratively maximize the heterogeneity of the average treatment effect across the strata (38). We defined the treatment as PTSD and the outcome as substance use, and we first tested a number of parameters to identify the best method for the causal forests given the data. This included testing a minimum node size of 5, 30, 50, and 100; testing the number of considered variables as default $\sqrt{p}+20$ , 10% of *p*, and 30% of *p* considered for a given split, with *p* defined as the number of variables in the full covariate matrix (p = 128); and testing the number of trees as 1,000 trees, 2,000 trees, and 5,000 trees per forest. We found no major differences in fit when using the default $\sqrt{p}+20$ , with 2,000 trees and five variables in the node size and proceeded with these parameters. We reported the variable importance for tobacco, alcohol, and cannabis use frequency outcomes. We generated variable importance using the *grf* package, based on the weighted counts of the proportion of splits on the variable of interest to a depth of 4 (39). We also used the causal forests similarly to generate doubly robust scores to stratify the risk as high versus low and to compare the conditional average treatment effect (CATE) between these strata.

In this way, the CATE can be used similarly to a propensity score. The most notable difference is that it was developed from the causal forest, which seeks to maximize the difference in the relationship between PTSD and substance use between the strata (rather than associated with only the exposure or outcome). Therefore, the CATE as a score represents a stratification tool that similarly maximizes the difference in the effect of PTSD and substance use. We used the median as the cutoff to stratify, theoretically, that the two strata are as maximally apart as the data indicate. We provide a sensitivity analysis using additional cutoff (25th and 75th, and 10th and 90th) percentiles of the CATE as a score in **Supplementary Table 4**.

We conducted an omnibus evaluation of the calibration by regressing the scores on the treatment effect, whereby a mean forest prediction nearing 1 indicated good calibration in the mean model and tested the statistical significance of a main treatment effect and a differential forest prediction nearing 1 indicated good calibration of the CATE model for both high/low score strata and tested the statistical significance of a differential treatment effect (39, 40).

### 2.5 Missingness

We assessed missingness and determined that it was likely to be missing at random (MAR) using graphical methods and t-tests for whether a variable was missing (yes/no) and our primary measures of the PCL-5 and frequency of substance use. We conducted multiple imputation by chained equations for 20 datasets across 30 iterations each. We pooled all model estimates using Rubin’s rules (41).

## 3 Results

In our sample, we had 2,943 participants overall, with 1,844 without PCL-5 symptoms indicative of likely PTSD diagnosis and 1,099 with PCL-5 symptom severity indicative of likely diagnosis at baseline, defined as a score of 33 or greater (**Table 1**). Participants without likely PTSD tended to be younger (mean = 34.8 years) compared to those with likely PTSD (mean = 37.7 years, t-test p-value<0.001). Overall, our sample was primarily non-Hispanic Black/African-American, with 49.5% (N = 1,458), followed by non-Hispanic White/European American (34.7%, n = 1,020) and Hispanic (11.6%, n = 342). The majority of our participants were female assigned at birth (61.8%, n = 1,818) and identified as cisgender female (61.7%, n = 1,815). Being cisgender female was overrepresented in our likely PTSD sample (70.2%, n = 772, p-value <0.001).

**Table 1:** Sociodemographic information and prevalent substance exposure stratified by likely PTSD diagnosis at emergency department recruitment visit.

	Unlikely PTSD ^1^ N = 1,844	Likely PTSD N = 1,099	Overall N = 2,943	p-Value ^2^
Age, mean (SD)	34.8 (13.1)	37.7 (13.5)	34.9 (11.3)	<0.001
Median (min, max)	31.5 (18.0, 74.0)	34.0 (18.0, 73.0)	32 (18, 74)	
Race/ethnicity				
Hispanic	207 (11.2%)	135 (12.3%)	342 (11.6%)	<0.001
Non-Hispanic Black	969 (52.5%)	489 (44.5%)	1,458 (49.5%)	
Non-Hispanic other	67 (3.6%)	44 (4.0%)	111 (3.8%)	
Non-Hispanic White	593 (32.2%)	427 (38.9%)	1,020 (34.7%)	
Missing	8 (0.4%)	4 (0.4%)	12 (0.4%)	
Sex assigned at birth				<0.001
Male	796 (43.2%)	328 (29.8%)	1,124 (38.2%)	
Female	1,047 (56.8%)	771 (70.2%)	1,818 (61.8%)	
Missing	1 (0.1%)	0	1 (0.0%)	
Gender identity				<0.001
Male	795 (43.1%)	325 (29.6%)	1,120 (38.1%)	
Female	1,043 (56.6%)	772 (70.2%)	1,815 (61.7%)	
Transgender	2 (0.1%)	2 (0.2%)	4 (0.1%)	
None	3 (0.2%)	0 (0%)	3 (0.1%)	
Missing	1 (0.1%)	0 (0%)	1 (0.0%)	
Marital status				0.002
Divorced	252 (13.7%)	198 (18.0%)	450 (15.3%)	
Married	375 (20.3%)	233 (21.2%)	608 (20.7%)	
Never married	1,175 (63.7%)	636 (57.9%)	1,811 (61.5%)	
Widowed	30 (1.6%)	27 (2.5%)	57 (1.9%)	
Missing	12 (0.7%)	5 (0.5%)	17 (0.6%)	
Education				<0.001
No HS	11 (0.6%)	4 (0.4%)	15 (0.5%)	
Some/finished HS	1,243 (67.4%)	661 (60.1%)	1,904 (64.7%)	
Some/finished college	476 (25.8%)	331 (30.1%)	807 (27.4%)	
Graduate school	108 (5.9%)	100 (9.1%)	208 (7.1%)	
Missing	6 (0.3%)	3 (0.3%)	9 (0.3%)	
Income				<0.001
≤$35,000/year	986 (53.5%)	658 (59.9%)	1,644 (55.9%)	
>$35,000 and ≤$75,000/year	349 (18.9%)	220 (20.0%)	569 (19.3%)	
>$75,000/year	224 (12.1%)	144 (13.1%)	368 (12.5%)	
Don’t know	285 (15.5%)	77 (7.0%)	362 (12.3%)	
Traumatic events				0.84
Assault	174 (9.5%)	113 (10.3%)	288 (9.8%)	
Collision	1,414 (76.7%)	833 (75.8%)	2,247 (76.4%)	
Fall	136 (7.4%)	77 (7.0%)	213 (7.2%)	
Other	118 (6.4%)	75 (6.8%)	193 (6.6%)	
Missing	1 (0.1%)	1 (0.1%)	2(0.1%)	
Tobacco				0.06
None	1,205 (65.3%)	679 (61.8%)	1,884 (64.0%)	
Any	632 (34.3%)	414 (37.7%)	1,046 (35.5%)	
Missing	7 (0.4%)	6 (0.5%)	13 (0.4%)	
Alcohol				0.10
None	721 (39.1%)	395 (35.9%)	1,116 (37.9%)	
Any	1,116 (60.5%)	699 (63.6%)	1,815 (61.7%)	
Missing	7 (0.4%)	5 (0.5%)	12 (0.4%)	
Cannabis				0.90
None	1,290 (70.0%)	774 (70.4%)	2,064 (70.1%)	
Any	539 (29.2%)	319 (29.0%)	858 (29.2%)	
Missing	15 (0.8%)	6 (0.5%)	21 (0.7%)	

^1^ Post-traumatic stress disorder (PTSD) was assessed using the PTSD Symptom Checklist for the DSM-5 (PCL-5) at emergency department (ED) baseline. Likely PTSD was defined as PCL-5 score greater than 33; a validated cutoff has been shown to indicate likely PTSD diagnosis based on the PCL-5 (26). Unlikely PTSD was defined as PCL-5 score of less than 33.

^2^ Continuous variables were tested for differences in means using two-sample t-tests assuming unequal variance. Categorical variables were tested for differences in distribution using chi-squared tests. Missing values were excluded from these bivariate tests.

Most of our participants had experienced a collision of some kind (primarily motor vehicle), representing 76.4% (n = 2,247) of our sample (**Supplementary Table 1**). There was no statistical evidence of a difference in index traumatic events between participants with likely PTSD and those without likely PTSD (p = 0.84). Notably, most participants reported alcohol use in the past 30 days prior to the ED visit (61.7%, n = 1,815) and were considered prevalent alcohol use cases, although this did not differ by PTSD symptoms (p = 0.10). Many also used cannabis in the prior 30 days (29.2%, n = 858) and/or smoked tobacco (35.5%, n = 1,046). These were defined as prevalent cannabis and prevalent tobacco groups, respectively. For incident use (defined as no use at baseline but later use at one or more subsequent timepoints), 162 (10.0% of those not smoking at baseline) participants were incident tobacco users, 296 (30.4% of those not drinking at baseline) were incident alcohol users, and 141 (7.9% of those not using cannabis at baseline) were incident cannabis users.

When examining substance use outcomes at week 8, month 3, month 6, and month 12 in the four-timepoint lagged model (**Table 2**), we found significant associations between PTSD symptoms and future tobacco use (incidence rate ratio (IRR): 1.003, 95% CI: 1.000, 1.005, p = 0.02) and alcohol use (IRR: 1.002, 95% CI: 1.000, 1.004, p = 0.03). There was no significant association with future cannabis use (IRR: 1.002, 95% CI: 0.999, 1.005, p = 0.13). PTSD symptoms were also associated with future quantity of tobacco smoked (IRR: 1.005, 95% CI: 1.001, 1.01) and quantity of alcohol consumed (IRR: 1.003, 95% CI: 1.001, 1.005). Similar patterns in the three-timepoint lagged model were demonstrated when we used only month 3, month 6, and month 12 time periods (**Supplementary Table 2**).

**Table 2:** Generalized estimating equations using Poisson model of post-traumatic stress symptoms and tobacco smoking frequency and quantity, alcohol use frequency and quantity, and cannabis frequency, controlling for sociodemographic factors using four timepoints.

	Tobacco frequency ^2^		Tobacco quantity ^3^		Alcohol frequency		Alcohol quantity ^3^		Cannabis frequency	
	Incidence rate (95% CI)	p-Value	Incidence Rate (95% CI)	p-Value	Incidence rate (95% CI)	p-Value	Incidence rate (95% CI)	p-Value	Incidence rate (95% CI)	p-Value
PTSD symptoms ^1^	1.003 (1, 1.005)	0.02	1.005 (1.001, 1.01)	0.01	1.002 (1.000, 1.004)	0.03	1.003 (1.001, 1.01)	0.001	1.002 (0.999, 1.005)	0.13
Time	0.966 (0.938, 0.996)	0.03	0.98 (0.93, 1.02)	0.29	0.99 (0.96, 1.012)	0.28	0.97 (0.94, 0.997)	0.03	0.988 (0.949, 1.029)	0.55
Marital status										
Married (Ref)										
Never married	1.09 (0.87, 1.36)	0.46	1.09 (0.87, 1.36)	0.46	1.15 (1.01, 1.3)	0.04	1.14 (1.01, 1.28)	0.03	1.42 (1.14, 1.78)	0.002
Divorced	1.46 (1.14, 1.88)	0.003	1.46 (1.14, 1.88)	0.003	0.997 (0.85, 1.17)	0.97	1.12 (0.98, 1.29)	0.11	1.35 (1.04, 1.75)	0.02
Widowed/other	1.002 (0.62, 1.61)	0.99	1.002 (0.62, 1.61)	0.99	1.2 (0.88, 1.64)	0.24	1.17 (0.88, 1.55)	0.29	1.48 (0.91, 2.38)	0.11
Gender										
Cisgender female (Ref)										
Cisgender male	1.48 (1.27, 1.72)	<0.001	1.48 (1.27, 1.72)	<0.001	1.18 (1.08, 1.3)	<0.001	1.25 (1.15, 1.36)	<0.001	1.35 (1.19, 1.53)	<0.001
Transgender	2.99 (0.67, 13.31)	0.15	2.99 (0.67, 13.31)	0.15	0.87 (0.43, 1.73)	0.68	1.31 (0.69, 2.46)	0.41	2.05 (0.77, 5.48)	0.15
Race/ethnicity										
Hispanic (Ref)										
Non-Hispanic Black	1.16 (0.97, 1.38)	0.10	0.7 (0.24, 2.03)	0.51	0.78 (0.37, 1.66)	0.52	0.75 (0.43, 1.32)	0.32	0.97 (0.79, 1.17)	0.72
Non-Hispanic other	1.02 (0.72, 1.45)	0.91	0.55 (0.18, 1.75)	0.31	0.68 (0.31, 1.51)	0.35	0.63 (0.35, 1.14)	0.13	0.8 (0.55, 1.16)	0.23
Non-Hispanic White	1.44 (1.2, 1.73)	<0.001	1.04 (0.35, 3.05)	0.94	0.86 (0.4, 1.83)	0.69	0.76 (0.43, 1.33)	0.33	1.04 (0.85, 1.28)	0.71
Age	0.998 (0.993, 1.003)	0.39	1.004 (0.997, 1.01)	0.25	0.999 (0.995, 1.003)	0.69	0.99 (0.99, 0.996)	<0.001	0.98 (0.98, 0.99)	<0.001
Income										
>$35k and ≤$75k (Ref)										
≤$35k	1.38 (1.18, 1.61)	<0.001	1.45 (1.17, 1.8)	<0.001	0.91 (0.8, 1.02)	0.12	0.95 (0.86, 1.06)	0.37	1.17 (0.97, 1.4)	0.09
>$75k	0.66 (0.51, 0.85)	0.002	0.46 (0.32, 0.67)	<0.001	1.34 (1.15, 1.56)	<0.001	1.11 (0.98, 1.26)	0.11	0.8 (0.6, 1.07)	0.13
Did not report	1.2 (0.99, 1.46)	0.07	1.24 (0.93, 1.64)	0.14	1.06 (0.91, 1.23)	0.45	0.97 (0.85, 1.11)	0.71	1.13 (0.9, 1.42)	0.30

^1^ Post-traumatic stress disorder (PTSD) symptoms were assessed via the PTSD Checklist for the DSM-5 (PCL-5) given at timepoints in week 2, week 8, month 3, and month 6 following an index traumatic event.

^2^ Substance use variables were recorded as past-month use, given at timepoints in week 8, month 3, month 6, and month 12 and lagged one timepoint ahead of the PTSD symptom assessments in order to maintain a prospective relationship between PTSD symptoms and substance use.

^3^ Quantity was defined as average amount used when a person was typically using. For tobacco, this was equivalent to average amount of cigarettes consumed, and for alcohol, this was equivalent to average number of drinks consumed. Cannabis quantity was not collected in the parent study and is not reported.

**Figure 1:**
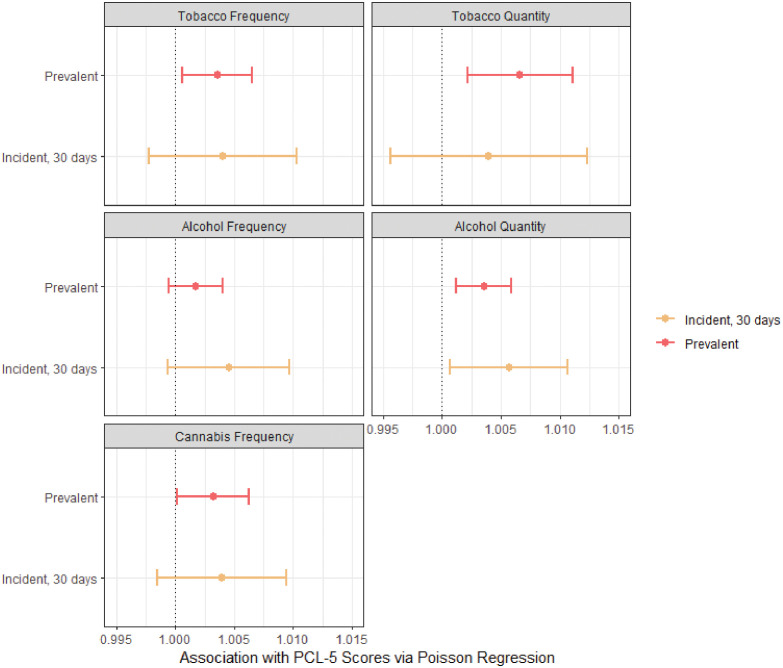
Comparison of incident *vs.* prevalent tobacco, alcohol, and cannabis use associations with PCL-5 scores for post-traumatic stress disorder (PTSD) symptoms using Poisson regression using three timepoints. Models were Poisson regression using generalized estimating equations with six timepoints for both PTSD exposures and substance use outcomes. Models were adjusted for all sociodemographic covariates and potential psychosocial confounders. Sociodemographic covariates included race/ethnicity (non-Hispanic Black, non-Hispanic White, Hispanic, and other non-Hispanic races/ethnicities), age, gender identity (male, female, and transgender), yearly income (<$35k/year, between $35k and $75k/year, >$75k/year, and “did not know”), and marital status (married, never married, and widowed/divorced/annulled). Psychosocial confounders included mindfulness scores, resiliency scores, emotional support scores, and chronic maximum stress scores. Prevalent and incident substance use were binarized: those who had previously used a substance in the past 30 days prior to the ED visit were considered prevalent, and those who had not were considered incident.

There were some differences between the effect of prevalent and incident substance use among individuals who did not use the given substance in the past 30 days prior to the emergency department baseline visit when examining substance use outcomes at month 3, month 6, and month 12 (**Figure 1**). Increased PTSD symptoms were associated with tobacco frequency (IRR: 1.004, 95% CI: 1.001, 1.01) and quantity (IRR: 1.01, 95% CI: 1.002, 1.01) among prevalent tobacco smokers but were not significantly associated among incident smokers who did not smoke at ED baseline (95% CI for frequency: 0.998, 1.01; 95% CI for quantity: 0.996, 1.01). Alcohol frequency did not show significant associations with PTSD, but alcohol quantity was associated with PTSD among both prevalent and incident drinkers. Incident alcohol consumption had a slightly higher point estimate association with increased PTSD symptoms compared to prevalent consumption (IRR: 1.004 *vs.* IRR: 1.003), although the respective confidence intervals overlapped. Cannabis showed a slight association among prevalent cases (IRR: 1.003, 95% CI: 1.0001, 1.01), although this was not significant for incident cannabis users.

**Figure 2:**
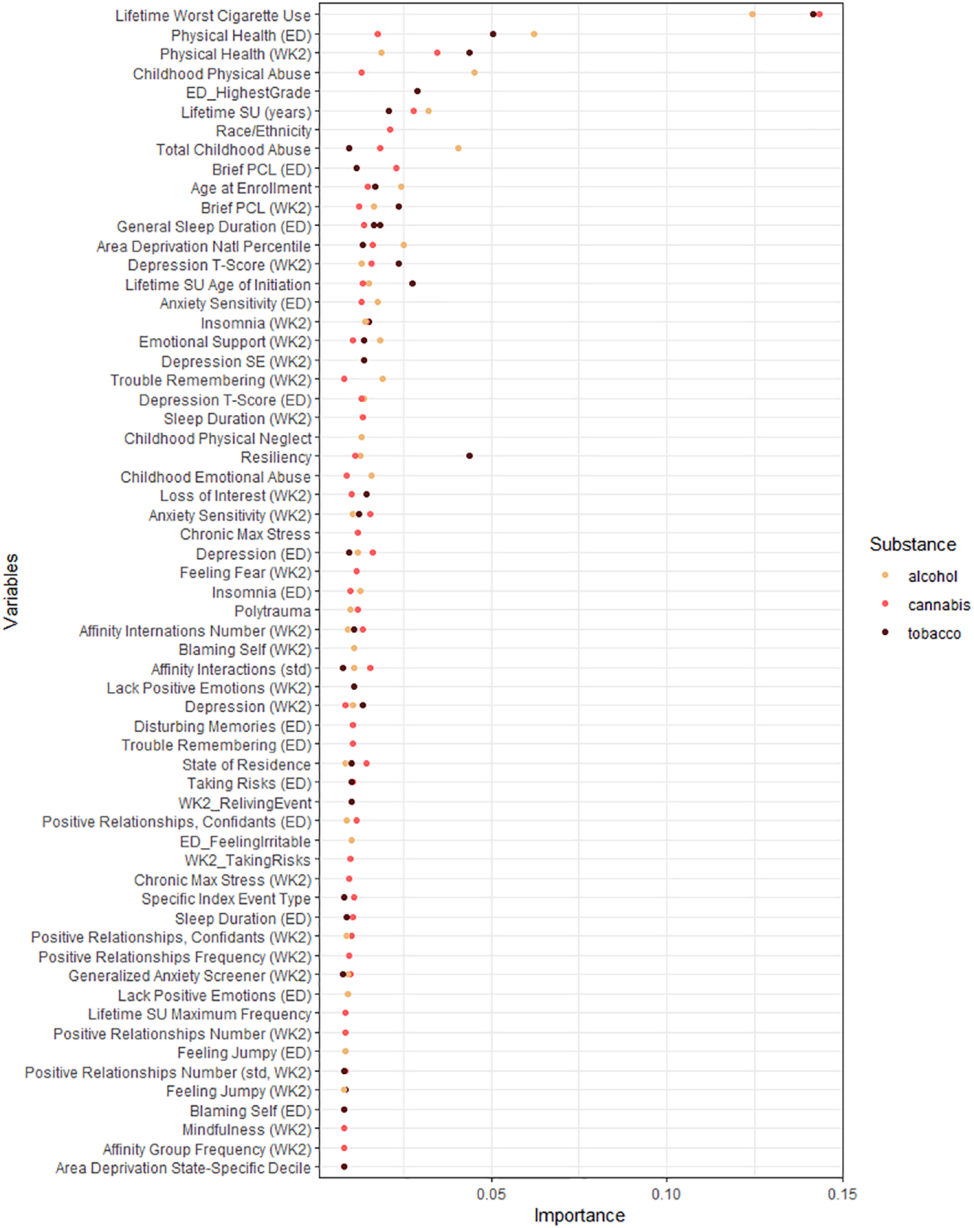
Variable importance for all moderators in the final causal forests predicting tobacco, alcohol, and cannabis use frequencies. Variable importance plot including all variables with greater than mean importance for their respective forest. Variable importance was generated using honest causal forests with 2,000 trees each. See **Supplementary Table 3** for description of relevant domain for included variables.

Across causal forests for tobacco, alcohol, and cannabis frequency using the three-timepoint lagging, we found that lifetime worst cigarette use was a major predictor of all causal forests (**Figure 2**). The SF-12, which measures overall health effects on quality of life, identified at baseline and week 2 was also important for all substances. For alcohol, lifetime physical abuse, the total score of the childhood trauma questionnaire, and lifetime years of substance use were the next most important factors. For tobacco, resiliency as measured by the CD-RISC-10 was the third most important predictor, and education was the fifth most important predictor, and they were not identified as important for either alcohol or cannabis. For cannabis, race and ethnicity were important stratification factors, which were not identified as important above the mean importance factor for the other substances.

Participants were stratified as low- and high-risk strata using the causal forests similar to propensity scores using the forest prediction probabilities for likely *vs.* unlikely substance use (**Table 3**). There was a significant difference in the conditional average treatment effect for tobacco frequency when stratifying low- and high-risk participants (difference in CATE: 0.03, 95% CI: 0.01, 0.06); however, this was not significant for alcohol (difference in CATE: 0.02, 95% CI: −0.001, 0.04) or cannabis (difference in CATE: 0.004, 95% CI: −0.01, 0.02). Bias estimates calculated as per Athey et al. (42) are reported in **Supplementary Figure 3**, which were all centered around the null.

**Table 3:** Estimated mean treatment effect and differential treatment effects using causal forests to maximize heterogeneity in the association between PTSD symptoms and tobacco, alcohol, and cannabis frequency of use.

	Mean forest effect ^1^	p-Value	Differential forest effect ^2^	p-Value	ATE among high strata	ATE among low strata	Difference in PTSD effect between strata
Tobacco	0.28	0.38	0.29	0.001	0.03	−0.01	0.03 (0.01, 0.06)
Alcohol	0.94	0.04	0.74	0.07	0.001	−0.02	0.02 (−0.0005, 0.04)
Cannabis	1.07	0.12	0.17	0.06	0.006	0.002	0.004 (−0.01, 0.02)

^1^ The mean forest effect demonstrates the overall estimated effect of all moderators in the causal forest on the average treatment effect (ATE).

^2^ The differential forest demonstrates their association with the difference by strata. Significant mean forest effects indicate that there is a significant overall effect of post-traumatic stress disorder (PTSD) on substance use when not stratifying (e.g., across all observations). Significant differential forest effects indicate that there is a statistically significant difference in the ATE among the high vs. low strata and indication of an interactive effect captured by the forest.

## 4 Discussion

Our study used a novel cohort of trauma-exposed adults to understand the impact of post-traumatic symptoms on substance use behaviors. We found that PTS symptom scores were highly associated with future tobacco and alcohol use after controlling for sociodemographic factors, but there was little evidence of a relationship with future cannabis use. We also found that prior substance use behaviors were the most important stratification factor of the risk of future substance use. Even for alcohol or cannabis frequency, lifetime reported worst cigarette use was the most important predictor, followed by self-rated overall health at ED and week 2 visits. Tobacco frequency demonstrated the greatest difference in the CATE between the strata determined by the causal forest-predicted probability. Our study has important implications for clinical providers to identify areas of intervention for patients who may have comorbid substance use and PTSD symptoms, as well as avenues for future research into these differential effects of PTSD on behavioral health.

Tobacco showed differences in prevalent use compared to incident use when modeling both frequency and quantity, suggesting that prevalent users are at the highest risk of changes in tobacco consumption with PTSD symptoms. Tobacco also demonstrated the largest CATE based on the causal forest stratification, although the calibration demonstrated that the fit may be insufficient in this causal forest, as demonstrated by the mean forest predictions and differential forest predictions both being less than 1. This fit was not improved when toggling parameters (such as number of trees and node size). Lifetime worst use of cigarettes (e.g., a higher number of cigarettes used when describing one’s most prolific lifetime period of smoking) was the most important variable for tobacco as well as alcohol and cannabis causal forests. Prior tobacco consumption behaviors and initiation make it more likely for participants to use substances again. Intervening on tobacco use may benefit the overall amelioration of substance use, although this should be investigated directly through experimental studies. As a potential area of intervention, while some treatment protocols for comorbid PTSD and tobacco dependence have described addressing both tobacco use and PTSD symptoms concurrently, these have not necessarily been tailored to stress response or the underlying causes of this association (43). This dual-treatment protocol has been demonstrated as effective in military populations (44); however, it has not been clear to what extent this is generalizable to civilian populations.

Overall health as measured by the 12-item Short Form Health Survey (32) and resiliency measured by the Connor–Davidson Resilience Scale-10 (28) were some of the highest importance variables for the relationship between PTSD symptoms and tobacco use. Given the physical effects of tobacco smoking over time, the importance of physical health may be related to the length of smoking. For example, individuals smoking for decades may experience worse health and also report higher past 30-day tobacco use compared to those who do not, and individuals experiencing higher rates of distress related to PTSD symptoms have also reported worse physical health in longitudinal studies. In one longitudinal study, there was evidence that PTSD symptoms mediate the relationship between traumatic event exposures (such as the number of traumatic events experienced) and poor physical health, as well as the number of traumatic events and substance use (45).

For alcohol quantity used, we saw consistent associations with the PCL-5 symptoms. In contrast, alcohol use frequency did not demonstrate consistent associations. The ATE was significant in the causal forests, suggesting that there was an overall effect of PTSD on alcohol quantity, but that there was no major differential effect in our sample, given that the CATE difference was not significant. This may be related to the rate of alcohol use in our sample, whereby it is not possible to differentiate between groups because the risk remains high overall. This may be related to selection bias in our sample, as many individuals presenting at the ED did so after a motor vehicle accident, potentially related to intoxicated driving. We did not directly ask participants if they were intoxicated during these accidents in order to reduce social desirability bias in baseline alcohol consumption responses, but future studies with more diverse traumatic index events in their sample may be better poised to identify relationships with alcohol. Notably, therefore, alcohol may precipitate the event that leads to PTSD symptoms, and those PTSD symptoms may precipitate future alcohol use. Multiple studies have identified that this relationship is likely to be bidirectional (46). It is hypothesized that different risk pathways may be involved in which develops first (47). For chronic pain, for instance, this is considered the “mutual maintenance” hypothesis, whereby distress and disability increase both chronic pain and PTSD, and a host of internal psychological factors further this relationship (48). No study to date has directly tested the mutual maintenance of alcohol use and PTSD symptoms; however, it stands to reason that a similar phenomenon can occur.

It was notable that physical abuse and childhood trauma variables were in the top 5 causal modifiers. Childhood trauma especially has been previously investigated as an exacerbating factor for alcohol dependence later in life (49, 50), and it is notable that this was more important than more proximate adult variables. This suggests that while there is evidence that facets such as mindfulness (51–53) or resiliency (12, 54) are related to alcohol use, these are unlikely to mitigate this relationship in our sample. However, in a high-risk sample, these moderating factors may have only slight effects overall. Future studies should investigate prior trauma in the context of other proximate psychological modifiers in samples with more diverse substance use behaviors.

Our study found no evidence that PTSD symptoms affected future cannabis use. This comports with a recent study from the AURORA cohort that there was little difference in the relationship between PTSD symptoms and groups already using cannabis (55). There is a growing interest in the therapeutic uses of cannabis for a range of mental health disorders, including PTSD, as evidenced by the increased legalization of cannabis in the United States. Notably, PTSD diagnosis is a qualifying condition for citizens to obtain a medical marijuana license within 29 states as of 2022 (56). Under this dynamic, we would expect that prior PTSD symptoms would increase future cannabis use if participants were treating their symptoms with cannabis. To this hypothesis, a systematic review of 10 papers found suggestive but overall low-quality evidence of cannabis’ effectiveness in reducing PTSD symptoms (57).

For instance, in a randomized controlled trial of different cannabis formulations, there was no difference in PTSD symptoms between cannabis treatment and placebo groups (58). However, in an observational study that observed participants for a year, they did report reduced PTSD symptoms associated with self-reported cannabis use (59). One of the largest studies of 2,276 veterans in intensive PTSD treatment programs found an increase in PTSD symptomology among those who started or continued to use cannabis after discharge from the treatment program (60). Our findings, however, represent a large civilian cohort not in intensive treatment programs, and therefore, more research should investigate the generalizability between our study and others and how individuals may relate to cannabis for therapeutic purposes.

Notably, the timing of cannabis use *vs.* PTSD symptoms is also critical in interpreting these findings. We lagged PTSD symptoms behind cannabis exposure in order to directly test a potential self-medication hypothesis, where one would expect increased prior PTSD symptoms associated with future cannabis use. If, instead, current cannabis use decreases PTSD symptoms, we would see a negative effect; in our sensitivity analysis using cross-sectional data (e.g., PTSD symptoms and cannabis use ascertained at the same timepoint), we continued to not see an association, however. This suggests that the association between PTSD symptoms and cannabis use may be subject to confounding or mediation by other factors in our sample that we have controlled for while other studies have not, or other studies may have over-adjusted models and opened epidemiological collider stratification biases not subject in our study. In *post-hoc* analyses, we had the power to detect reasonable effect sizes, which suggests that it is unlikely our findings represent a false negative alone.

Our findings demonstrate that different substances may have different relationships with PTSD symptoms. This comports with and expands upon prior findings (61–63). For example, a 2018 study found that different symptom types for PTSD had specific associations, alcohol use was associated with avoidance symptoms, and hyperarousal symptoms were elevated among participants with drug use disorders (64). It further stands to theoretically reason that, due to different subjective effects of substances, those with hyperarousal symptoms primarily may be drawn to a particularly depressant substance that results in greater feelings of numbing or hypoarousal, while those with avoidant symptoms may seek another subjective response. While our study aggregated across symptom dimensions using a validated questionnaire, future studies may select specific dimensions of PTSD symptoms and their associations with substance use behaviors. This would provide information that could personalize intervention recommendations for patients even further.

In examining psychosocial factors, which are often the basis for interventions (e.g., mindfulness and meditation training techniques, and recommendations for building supportive relationships to mitigate symptoms), we found that they did not demonstrate the most important associations. Rather, areas of general health, childhood traumatic events, and even sleep quality showed some of the highest associations before areas such as resiliency and mindfulness. This suggests that simply building cognitive or psychological resiliency may be better supported with concurrent physical health considerations and sleep protocols. Physical wellbeing has been previously identified as an important resilience factor (65–67), as well as larger sociodemographic factors including income and education (51, 54, 68, 69). Our findings highlight the holistic nature of trauma and substance use, and their effects on the body and behavior cannot be ignored in favor of a purely mental understanding.

Our use of causal forests also demonstrates a novel approach to investigating effect modification. With a large cohort, we have a sufficient sample size to fit the generalized random forest algorithm and estimate conditional average treatment effects. To our knowledge, only one study to date has previously applied this method related to post-traumatic stress, which focused on a prospective cohort of older Japanese adults after the 2011 Great East Japan Earthquake (70). This paper may not be generalizable to other populations who have not experienced a single mass-casualty incident, and they used 51 predictors of the CATE, compared to our 128. Using a “bottom-up” approach may identify potential effects that would not be investigated from a “top-down” theory-driven approach alone based on established literature. Notably, the use of forests is similar to allowing for multi-order interactions (such as three-way and four-way) based on the splitting algorithm; however, by aggregating over thousands of forests, it reduces issues of overfitting that may otherwise arise (39). However, it is not without its limits: as a non-parametric estimation, it may have issues with confidence interval construction based on undersmoothing versus a bias-correction method and, similar to more standard methods, experience edge effects in slope estimates.

Our study had a number of limitations, including its reliance primarily on self-report. Toxicological testing was not available for participants at the time of their recruitment, and we relied on self-reported questionnaires, which may be subject to social desirability bias (71). The majority of our participants were recruited following a motor vehicle accident in which they were the driver, which may reduce the number of substance use endorsements in our sample, which would bias our findings toward null hypotheses. Future research may consider toxicological testing at recruitment and follow-up via urine or hair samples to ascertain outcomes. Despite this, we still reported high levels of substance use, which suggests that many did feel comfortable reporting their past 30-day use.

While our sample included many different index traumatic events for eligibility, the majority of our sample represented motor vehicle accident cases, which may not be generalizable to samples with other traumatic events, such as mass casualty events or assaults. This was likely related to our recruitment strategy from EDs, and future studies may consider sampling strategies that prioritize other trauma-exposed adults to understand whether these findings replicate in these additional samples. Our sample is unlikely to reflect individuals who do not experience a traumatic event resulting in physical injury, although our PTSD assessment did not require that symptoms be solely related to the index traumatic event. Therefore, while our PTSD metric may capture some effect of other traumatic events that participants may have experienced, this remains a limitation of the sample and our findings.

There also may be measurement bias in our definition of PTSD and other psychosocial metrics. Given known limitations in the DSM-5 (72) and other measures (73), which may not capture all dimensions of PTSD, depression, anxiety, or other constructs that we considered in our causal forest analysis, our findings may be biased. While we examined many factors, there may be additional unmeasured confounding. Finally, we were limited by the sample size of other substances and were unable to investigate whether these findings held also for opioids, stimulants, etc.

Our study also had numerous strengths; primarily, the cohort represents a national civilian population with a range of psychosocial data and prospective follow-ups. Prior longitudinal cohorts investigating both PTSD and SU to date have been made up of participants primarily in treatment for PTSD or SU (74–76) or veteran samples (77, 78). This allowed us to understand the effect of these various constructs over time and track changes in the frequency of use with greater granularity than had we been limited to binarized variables. We also considered the three most popular substances in the United States in the same sample, allowing for comparisons to be made between substances. Finally, we were able to include the largest reported number of psychosocial factors as potential modifiers using a novel statistical method, which allowed for a comprehensive understanding of our participants compared to smaller cohorts.

The public health implications of this work may benefit directing relevant interventions to patients with the highest risk of PTSD symptoms and future substance use behaviors. We identified prior substance use behaviors, overall health, and childhood traumatic events as highly important. Future studies should consider integrating these screening factors and examine if they can effectively stratify risk in a clinical setting to benefit patient outcomes. Identifying people at risk early may direct interventions for primary and secondary prevention against future or worsening substance use after a traumatic event. While informing patients they should be careful of any substance behaviors, providing insight into specific substances may better address their needs beyond generalized advice.

## 5 Conclusion

We demonstrated differential effects of PTSD on future tobacco use using causal forest modeling, with prior cigarette use being the most important factor. We also found that future alcohol use increases with increased PTSD symptoms. Finally, we found that future cannabis frequency of use did not demonstrate a consistent relationship with PTSD symptoms, suggesting that there may not be a clear association when controlling for potential confounders or when accounting for multiple moderators. Taken together, these findings show that substance use following a traumatic event can vary based on personal substance use histories and depending on how the substance use itself is measured. This has important implications both for future epidemiological research that may consider different metrics of substance use and for clinicians interested in identifying exacerbating factors that may moderate this relationship in their patient populations.

In the future, we recommend similar investigations with sample sizes spanning a wider range of traumatic events that would allow for sufficient subgroup analyses. In our sample, the focus on motor vehicle incidents may limit generalizability or obscure event-specific relationships with substance use behaviors. We also recommend studies that examine differences between substance use disorders and subclinical behaviors and subscale-specific PTSD evaluations; this would essentially create a matrix of potential facets of PTSD that then may be associated with subclinical *vs.* clinical disordered substance use, providing even more tailored insights. Future work should build upon findings of the most important variables to develop risk stratification methods that may eventually aid in personalized recommendations to patients following a traumatic event to mitigate their individual substance use risks.

## Data availability statement

The datasets analyzed for this study can be found in the NIMH data archive [https://nda.nih.gov/edit_collection.html?id=2526].

## Ethics statement

The studies involving humans were approved by Harvard Longwood Campus Institutional Review Board. The studies were conducted in accordance with the local legislation and institutional requirements. Written informed consent for participation was not required from the participants or the participants’ legal guardians/next of kin in accordance with the national legislation and institutional requirements.

## Author contributions

HG-D and CD identified the primary research question of interest and defined the initial analytic plan. HG-D completed all analyses for tables and figures and wrote the initial draft of the manuscript. CD, JM, SL, and KK provided feedback on analyses throughout its initial stages and provided writing for the manuscript. All authors contributed to the article and approved the submitted version.

## References

[1] Benjet C Bromet E Karam EG Kessler RC McLaughlin KA Ruscio AM. The epidemiology of traumatic event exposure worldwide: results from the World Mental Health Survey Consortium. Psychol Med (2016) 46(2):327–43. doi: 10.1017/S0033291715001981 PMC4869975 26511595

[2] McLaughlin KA Koenen KC Friedman MJ Ruscio AM Karam EG Shahly V. Sub-threshold post traumatic stress disorder in the WHO world mental health surveys. Biol Psychiatry (2015) 77(4):375–84. doi: 10.1016/j.biopsych.2014.03.028 PMC4194258 24842116

[3] Koenen KC Ratanatharathorn A Ng L McLaughlin KA Bromet EJ Stein DJ. Posttraumatic stress disorder in the World Mental Health Surveys. Psychol Med (2017) 47(13):2260–74. doi: 10.1017/S0033291717000708 PMC6034513 28385165

[4] Conway KP Compton W Stinson FS Grant BF. Lifetime comorbidity of DSM-IV mood and anxiety disorders and specific drug use disorders: results from the National Epidemiologic Survey on Alcohol and Related Conditions. J Clin Psychiatry (2006) 67(2):247–57. doi: 10.4088/JCP.v67n0211 16566620

[5] Stewart SH Conrod PJ. Psychosocial models of functional associations between posttraumatic stress disorder and substance use disorder. In: Trauma and substance abuse: Causes, consequences, and treatment of comorbid disorders. Washington, DC, US: American Psychological Association (2003). p. 29–55.

[6] Ullman SE Relyea M Peter-Hagene L Vasquez AL. Trauma histories, substance use coping, PTSD, and problem substance use among sexual assault victims. Addictive Behaviors (2013) 38(6):2219–23. doi: 10.1016/j.addbeh.2013.01.027 PMC3622163 23501138

[7] Chilcoat HD Breslau N. Investigations of causal pathways between ptsd and drug use disorders. Addictive Behaviors (1998) 23(6):827–40. doi: 10.1016/S0306-4603(98)00069-0 9801719

[8] Kearns NT Blumenthal H Contractor AA Aston ER Metrik J. Effect of trauma-related stress after alcohol consumption on perceived likelihood of negative consequences and willingness to drive. Addict Behav (2021) 117:106836. doi: 10.1016/j.addbeh.2021.106836 33529850 PMC7956021

[9] Nazarian D Kimerling R Frayne SM. Posttraumatic stress disorder, substance use disorders, and medical comorbidity among returning U. S veterans J Traumatic Stress (2012) 25(2):220–5. doi: 10.1002/jts.21690 22522739

[10] Acierno R Kilpatrick DG Resnick H Saunders B De Arellano M Best C. Assault, PTSD, family substance use, and depression as risk factors for cigarette use in youth: findings from the national survey of adolescents. J Trauma Stress. (2000) 13(3):381–96. doi: 10.1023/A:1007772905696 10948480

[11] Price RK Risk NK Haden AH Lewis CE Spitznagel EL. Post-traumatic stress disorder, drug dependence, and suicidality among male Vietnam veterans with a history of heavy drug use. Drug Alcohol Dependence (2004) 76:S31–43. doi: 10.1016/j.drugalcdep.2004.08.005 15555815

[12] Wingo AP Ressler KJ Bradley B. Resilience characteristics mitigate tendency for harmful alcohol and illicit drug use in adults with a history of childhood abuse: A cross-sectional study of 2024 inner-city men and women. J Psychiatr Res (2014) 51:93–9. doi: 10.1016/j.jpsychires.2014.01.007 PMC4605671 24485848

[13] Astin MC Lawrence KJ Foy DW. Posttraumatic stress disorder among battered women: risk and resiliency factors. Violence Vict (1993) 8(1):17–28. 8292561

[14] Brady KT McCauley JL Back SE. The comorbidity of post-traumatic stress disorder (PTSD) and substance use disorders. In: el-Guebaly N Carrà G Galanter M Baldacchino AM, editors. Textbook of Addiction Treatment: International Perspectives. Cham: Springer International Publishing (2021). p. 1327–39. doi: 10.1007/978-3-030-36391-8_93

[15] Davis JP Diguiseppi G De Leon J Prindle J Sedano A Rivera D. Understanding pathways between PTSD, homelessness, and substance use among adolescents. Psychol Addictive Behaviors (2019) 33(5):467–76. doi: 10.1037/adb0000488 31343198

[16] Williamson L Dell CA Osgood N Charlmers D Lohnes C Carleton RN. Examining Changes in Posttraumatic Stress Disorder Symptoms and Substance Use Among a Sample of Canadian Veterans Working with Service Dogs: An Exploratory Patient- Oriented Longitudinal Study (2021). Available at: https://ourspace.uregina.ca/handle/10294/15595.

[17] Ouimette P Wade M Coolhart D Tirone V Goodwin E Semenec S. Measuring PTSD course among substance use disorder patients: A pilot study of the interrater reliability and validity of the longitudinal interval follow-up evaluation (LIFE). Traumatol (2010) 16(3):19–26. doi: 10.1177/1534765610368570

[18] Livingston NA Lee DJ Mahoney CT Farmer SL Cole T Marx BP. Longitudinal assessment of PTSD and illicit drug use among male and female OEF-OIF veterans. Addictive Behaviors (2021) 118:106870. doi: 10.1016/j.addbeh.2021.106870 33667852 PMC9020386

[19] Tyler Boden M Kimerling R Kulkarni M Bonn-Miller MO Weaver C Trafton J. Coping among military veterans with PTSD in substance use disorder treatment. J Subst Abuse Treat (2014) 47(2):160–7. doi: 10.1016/j.jsat.2014.03.006 24854218

[20] Kline A Weiner MD Ciccone DS Interian A LSt H Losonczy M. Increased risk of alcohol dependency in a cohort of National Guard troops with PTSD: A longitudinal study. J Psychiatr Res (2014) 50:18–25. doi: 10.1016/j.jpsychires.2013.11.007 24332924

[21] Mills KL Teesson M Ross J Peters L. Trauma, PTSD, and substance use disorders: findings from the Australian national survey of mental health and well-being. AJP (2006) 163(4):652–8. doi: 10.1176/ajp.2006.163.4.652 16585440

[22] Hedtke KA Ruggiero KJ Fitzgerald MM Zinzow HM Saunders BE Resnick HS. A longitudinal investigation of interpersonal violence in relation to mental health and substance use. J Consulting Clin Psychol (2008) 76(4):633–47. doi: 10.1037/0022-006X.76.4.633 18665691

[23] Pericot-Valverde I Elliott RJ Miller ME Tidey JW Gaalema DE. Posttraumatic stress disorder and tobacco use: A systematic review and meta-analysis. Addictive Behaviors (2018), 84:238–47. doi: 10.1016/j.addbeh.2018.04.024 PMC7285418 29753221

[24] McLean SA Ressler K Koenen KC Neylan T Germine L Jovanovic T. The AURORA study: A longitudinal, multimodal library of brain biology and function after traumatic stress exposure. Mol Psychiatry (2020) 25(2):283–96. doi: 10.1038/s41380-019-0581-3 PMC6981025 31745239

[25] Weathers F Blake D Schnurr P Kaloupek D Marx B Keane T. The Life Events Checklist for DSM-5 (LEC-5). Washington DC: National Center for PTSD (2013). Available at: www.ptsd.va.gov.

[26] Bovin MJ Marx BP Weathers FW Gallagher MW Rodriguez P Schnurr PP. Psychometric properties of the PTSD Checklist for Diagnostic and Statistical Manual of Mental Disorders-Fifth Edition (PCL-5) in veterans. Psychol Assess (2016) 28(11):1379–91. doi: 10.1037/pas0000254 26653052

[27] Hamilton CM Strader LC Pratt JG Maiese D Hendershot T Kwok RK. The PhenX Toolkit: get the most from your measures. Am J Epidemiol (2011) 174(3):253–60. doi: 10.1093/aje/kwr193 PMC3141081 21749974

[28] Connor KM Davidson JRT. Development of a new resilience scale: the Connor-Davidson Resilience Scale (CD-RISC). Depress Anxiety (2003) 18(2):76–82. doi: 10.1002/da.10113 12964174

[29] Baer RA Smith GT Hopkins J Krietemeyer J Toney L. Using self-report assessment methods to explore facets of mindfulness. Assessment (2006) 13(1):27–45. doi: 10.1177/1073191105283504 16443717

[30] Cella D Yount S Rothrock N Gershon R Cook K Reeve B. The patient-reported outcomes measurement information system (PROMIS). Med Care (2007) 45(5 Suppl 1):S3–11. doi: 10.1097/01.mlr.0000258615.42478.55 PMC2829758 17443116

[31] Amtmann D Kim J Chung H Bamer AM Askew RL Wu S. Comparing CESD-10, PHQ-9, and PROMIS depression instruments in individuals with multiple sclerosis. Rehabil Psychol (2014) 59:220–9. doi: 10.1037/a0035919 PMC4059037 24661030

[32] Stewart A Sherbourne CD Ware JE Hays RD Wells KB Berry SH. Measuring Functioning and Well-Being: The Medical Outcomes Study Approach. Durham, NC: Duke University Press (1992). Available at: https://www.rand.org/pubs/commercial_books/CB361.html.

[33] Bernstein DP Stein JA Newcomb MD Walker E Pogge D Ahluvalia T. Development and validation of a brief screening version of the Childhood Trauma Questionnaire. Child Abuse Negl (2003) 27(2):169–90. doi: 10.1016/S0145-2134(02)00541-0 12615092

[34] Bernstein DP Fink L Handelsman L Foote J Lovejoy M Wenzel K. Initial reliability and validity of a new retrospective measure of child abuse and neglect. Am J Psychiatry (1994) 151(8):1132–6. doi: 10.1176/ajp.151.8.1132 8037246

[35] Krause N Borawski-Clark E. Social class differences in social support among older adults1. Gerontologist (1995) 35(4):498–508. doi: 10.1093/geront/35.4.498 7557520

[36] Buysse DJ Reynolds CF Monk TH Berman SR Kupfer DJ. The Pittsburgh sleep quality index: A new instrument for psychiatric practice and research. Psychiatry Res (1989) 28(2):193–213. doi: 10.1016/0165-1781(89)90047-4 2748771

[37] University of Wisconsin School of Medicine and Public Health. Area Deprivation Index 2019 (2019). Available at: https://www.neighborhoodatlas.medicine.wisc.edu/.

[38] Jawadekar N Kezios K Odden MC Stingone JA Calonico S Rudolph K. Practical guide to honest causal forests for identifying heterogeneous treatment effects. Am J Epidemiol (2023) 192:kwad043. doi: 10.1093/aje/kwad043 36843042

[39] Athey S Tibshirani J Wager S. Generalized random forests. Ann Statistics (2019) 47(2):1148–78. doi: 10.1214/18-AOS1709

[40] Athey S Wager S. Estimating treatment effects with causal forests: an application. Observational Stud (2019) 5(2):37–51. doi: 10.1353/obs.2019.0001

[41] Rubin DB. Multiple Imputation for Nonresponse in Surveys. New York, NY: John Wiley & Sons (1987). 326 p.

[42] Athey S Imbens G Pham T Wager S. Estimating average treatment effects: supplementary analyses and remaining challenges. Am Economic Review (2017) 107(5):278–81. doi: 10.1257/aer.p20171042

[43] Kelly MM Jensen KP Sofuoglu M. Co-occurring tobacco use and posttraumatic stress disorder: Smoking cessation treatment implications. Am J Addictions (2015) 24(8):695–704. doi: 10.1111/ajad.12304 26584242

[44] McFall M Saxon AJ Malte CA Chow B Bailey S Baker DG. Integrating tobacco cessation into mental health care for posttraumatic stress disorder: A randomized controlled trial. JAMA (2010) 304(22):2485–93. doi: 10.1001/jama.2010.1769 PMC4218733 21139110

[45] Del Gaizo AL Elhai JD Weaver TL. Posttraumatic stress disorder, poor physical health and substance use behaviors in a national trauma-exposed sample. Psychiatry Res (2011) 188(3):390–5. doi: 10.1016/j.psychres.2011.03.016 21481478

[46] Tripp JC Worley MJ Straus E Angkaw AC Trim RS Norman SB. Bidirectional relationship of posttraumatic stress disorder (PTSD) symptom severity and alcohol use over the course of integrated treatment. Psychol Addictive Behaviors (2020) 34(4):506–11. doi: 10.1037/adb0000564 PMC7266724 32105112

[47] Berenz EC Roberson-Nay R Latendresse SJ Mezuk B Gardner CO Amstadter AB. Posttraumatic stress disorder and alcohol dependence: Epidemiology and order of onset. psychol Trauma: Theory Res Practice Policy (2017) 9(4):485–92. doi: 10.1037/tra0000185 PMC5346471 27617659

[48] Sharp TJ Harvey AG. Chronic pain and posttraumatic stress disorder: mutual maintenance? Clin Psychol Rev (2001) 21(6):857–77. doi: 10.1016/S0272-7358(00)00071-4 11497210

[49] Brady KT Back SE. Childhood trauma, posttraumatic stress disorder, and alcohol dependence. Alcohol Res (2012) 34(4):408–13. doi: 10.1016/j.addbeh.2009.05.004 PMC3860395 23584107

[50] Stewart SH. Alcohol abuse in individuals exposed to trauma: A critical review. psychol Bulletin (1996) 120:83–112. doi: 10.1037/0033-2909.120.1.83 8711018

[51] Smith BW Ortiz JA Steffen LE Tooley EM Wiggins KT Yeater EA. Mindfulness is associated with fewer PTSD symptoms, depressive symptoms, physical symptoms, and alcohol problems in urban firefighters. J Consulting Clin Psychol (2011) 79:613–7. doi: 10.1037/a0025189 21875175

[52] Davis JP Pedersen ER Borsari B Bowen S Owen J Sedano A. Development of a mobile mindfulness smartphone app for post-traumatic stress disorder and alcohol use problems for veterans: Beta test results and study protocol for a pilot randomized controlled trial. Contemp Clin Trials (2023) 129:107181. doi: 10.1016/j.cct.2023.107181 37059261 PMC10225328

[53] Fernandez AC Wood MD Stein LAR Rossi JS. Measuring mindfulness and examining its relationship with alcohol use and negative consequences. Psychol Addictive Behaviors (2010) 24:608–16. doi: 10.1037/a0021742 PMC3924566 21198223

[54] Kim JI Park H Kim JH. The mediation effect of PTSD, perceived job stress and resilience on the relationship between trauma exposure and the development of depression and alcohol use problems in Korean firefighters: A cross-sectional study. J Affect Disord (2018) 229:450–5. doi: 10.1016/j.jad.2017.12.055 29331707

[55] Hinojosa CA Liew A An X Stevens JS Basu A van Rooij SJH. Associations of alcohol and cannabis use with change in posttraumatic stress disorder and depression symptoms over time in recently trauma-exposed individuals. psychol Med (2023) 54:1–12. doi: 10.1017/S0033291723001642 37309917 PMC10716364

[56] Project MP. MPP. PTSD and Medical Cannabis Programs. Available at: https://www.mpp.org/issues/medical-marijuana/ptsd-medical-cannabis-programs/.

[57] Hindocha C Cousijn J Rall M Bloomfield MAP. The effectiveness of cannabinoids in the treatment of posttraumatic stress disorder (PTSD): A systematic review. J Dual Diagnosis (2020) 16(1):120–39. doi: 10.1080/15504263.2019.1652380 31479625

[58] Bonn-Miller MO Sisley S Riggs P Yazar-Klosinski B Wang JB Loflin MJE. The short-term impact of 3 smoked cannabis preparations versus placebo on PTSD symptoms: A randomized cross-over clinical trial. PloS One (2021) 16(3):e0246990. doi: 10.1371/journal.pone.0246990 33730032 PMC7968689

[59] Bonn-Miller MO Brunstetter M Simonian A Loflin MJ Vandrey R Babson KA. The long-term, prospective, therapeutic impact of cannabis on post-traumatic stress disorder. Cannabis Cannabinoid Res (2022) 7(2):214–23. doi: 10.1089/can.2020.0056 PMC9070744 33998874

[60] Wilkinson ST Stefanovics E Rosenheck RA. Marijuana use is associated with worse outcomes in symptom severity and violent behavior in patients with posttraumatic stress disorder. J Clin Psychiatry (2015) 76(9):1174–80. doi: 10.4088/JCP.14m09475 PMC6258013 26455669

[61] Khoury L Tang YL Bradley B Cubells JF Ressler KJ. Substance use, childhood traumatic experience, and Posttraumatic Stress Disorder in an urban civilian population. Depression Anxiety (2010) 27(12):1077–86. doi: 10.1002/da.20751 PMC3051362 21049532

[62] Avant EM Davis JL Cranston CC. Posttraumatic stress symptom clusters, trauma history, and substance use among college students. J Aggression Maltreatment Trauma (2011) 20:539–555. doi: 10.1080/10926771.2011.588153

[63] McFall ME Mackay PW Donovan DM. Combat-related posttraumatic stress disorder and severity of substance abuse in Vietnam veterans. J Stud Alcohol (1992) 53(4):357–63. doi: 10.15288/jsa.1992.53.357 1619930

[64] Dworkin ER Wanklyn S Stasiewicz PR Coffey SF. PTSD symptom presentation among people with alcohol and drug use disorders: Comparisons by substance of abuse. Addictive Behaviors (2018) 76:188–94. doi: 10.1016/j.addbeh.2017.08.019 PMC5614876 28846939

[65] Bonanno GA Galea S Bucciarelli A Vlahov D. What predicts psychological resilience after disaster? The role of demographics, resources, and life stress. J Consulting Clin Psychol (2007) 75(5):671–82. doi: 10.1037/0022-006X.75.5.671 17907849

[66] Isaacs K Mota NP Tsai J Harpaz-Rotem I Cook JM Kirwin PD. Psychological resilience in U.S. military veterans: A 2-year, nationally representative prospective cohort study. J Psychiatr Res (2017) 84:301–9. doi: 10.1016/j.jpsychires.2016.10.017 27814502

[67] Agaibi CE Wilson JP. Trauma, PTSD, and resilience: A review of the literature. Trauma Violence Abuse (2005) 6(3):195–216. doi: 10.1177/1524838005277438 16237155

[68] El-Gabalawy R Blaney C Tsai J Sumner JA Pietrzak RH. Physical health conditions associated with full and subthreshold PTSD in U.S. military veterans: Results from the National Health and Resilience in Veterans Study. J Affect Disord (2018) 227:849–53. doi: 10.1016/j.jad.2017.11.058 PMC6269149 29689700

[69] Averill LA Smith NB Holens PL Sippel LM Bellmore AR Mota NP. Sex differences in correlates of risk and resilience associated with military sexual trauma. J Aggression Maltreatment Trauma (2019) 28(10):1199–215. doi: 10.1080/10926771.2018.1522408

[70] Shiba K Daoud A Kino S Nishi D Kondo K Kawachi I. Uncovering heterogeneous associations of disaster-related traumatic experiences with subsequent mental health problems: A machine learning approach. Psychiatry Clin Neurosciences (2022) 76(4):97–105. doi: 10.1111/pcn.13322 PMC9102396 34936171

[71] Latkin CA Edwards C Davey-Rothwell MA Tobin KE. The relationship between social desirability bias and self-reports of health, substance use, and social network factors among urban substance users in Baltimore, Maryland. Addictive Behaviors (2017) 73:133–6. doi: 10.1016/j.addbeh.2017.05.005 PMC5519338 28511097

[72] Pai A Suris AM North CS. Posttraumatic stress disorder in the DSM-5: controversy, change, and conceptual considerations. Behav Sci (Basel) (2017) 7(1):7. doi: 10.3390/bs7010007 28208816 PMC5371751

[73] Badger T Heitkemper M Lee K Bruner DW. The experience of PROMIS: pros and cons and unanswered questions. Nurs Outlook (2014) 62(5):332–8. doi: 10.1016/j.outlook.2014.06.009 PMC4167407 25218082

[74] Driessen M Schulte S Luedecke C Schaefer I Sutmann F Ohlmeier M. Trauma and PTSD in patients with alcohol, drug, or dual dependence: A multi-center study. Alcohol: Clin Exp Res (2008) 32(3):481–8. doi: 10.1111/j.1530-0277.2007.00591.x 18215214

[75] Tripp JC Jones JL Back SE Norman SB. Dealing with complexity and comorbidity: comorbid PTSD and substance use disorders. Curr Treat Options Psych (2019) 6(3):188–97. doi: 10.1007/s40501-019-00176-w

[76] Jacobsen LK Southwick SM Kosten TR. Substance use disorders in patients with posttraumatic stress disorder: A review of the literature. AJP (2001) 158(8):1184–90. doi: 10.1176/appi.ajp.158.8.1184 11481147

[77] Teeters JB Lancaster CL Brown DG Back SE. Substance use disorders in military veterans: prevalence and treatment challenges. Subst Abuse Rehabilitation (2017) 8:69–77. doi: 10.2147/SAR.S116720 PMC5587184 28919834

[78] McGuire AP Mota NP Sippel LM Connolly KM Lyons JA. Increased resilience is associated with positive treatment outcomes for veterans with comorbid PTSD and substance use disorders. J Dual Diagnosis (2018) 14(3):181–6. doi: 10.1080/15504263.2018.1464237 29668364

