# Supplementary material for: Post-traumatic stress and future substance use outcomes: leveraging antecedent factors to stratify risk
Source: Front Psychiatry. 2024 Mar 8;15:1249382. doi: 10.3389/fpsyt.2024.1249382 (PMC10957776; doi:10.3389/fpsyt.2024.1249382)
Supplement: Supplementary file 1 [file DataSheet_1.docx]

Supplementary Material

**Post-traumatic stress and future substance use outcomes: leveraging antecedent factors to stratify risk**

**Henri M Garrison-Desany^1^; Christy A Denckla^1^; Jacquelyn L Meyers^2^; Sarah D Linnstaedt^3^; Karestan C Koenen^4^; Stacey L House^5^; Francesca L Beaudoin^6,7^; Xinming An^3^; Jennifer S Stevens^8^; Donglin Zeng^9^; Thomas C Neylan^10^; Gari D Clifford^11,12^; Tanja Jovanovic^13^; Laura T Germine^14,15,16^; Kenneth A Bollen^17^; Scott L Rauch^14,18,16^; John P Haran^19^; Alan B Storrow^20^; Christopher Lewandowski^21^; Paul I Musey Jr.^22^; Phyllis L Hendry^23^; Sophia Sheikh^23^; Christopher W Jones^24^; Brittany E Punches^25^; Robert A Swor^26^; Nina T Gentile^27^; Vishnu P Murty^28^; Lauren A Hudak^29^; Jose L Pascual^30,31^; Mark J Seamon^32,31^; Erica Harris^33^; Anna M Chang^34^; Claire Pearson^35^; David A Peak^36^; Roland C Merchant^37^; Robert M Domeier^38^; Niels K Rathlev^39^; Brian J O'Neil^40^; Paulina Sergot^41^; Leon D Sanchez^37,42^; Steven E Bruce^43^; Jutta Joormann^44^; Steven E Harte^45,46^; Samuel A McLean^47,48^; Kerry J Ressler^50,16^**

*** Correspondence:** Henri M. Garrison-Desany; [hgarrisondesany@hsph.harvard.edu](mailto:hgarrisondesany@hsph.harvard.edu)

# Supplementary Figures and Tables

**Supplemental Figure 1.** Principal component analysis of past 30 day use frequency variables for tobacco, alcohol, cannabis, opiates, cocaine, hallucinogens, barbiturates, stimulants, and sedatives.

**
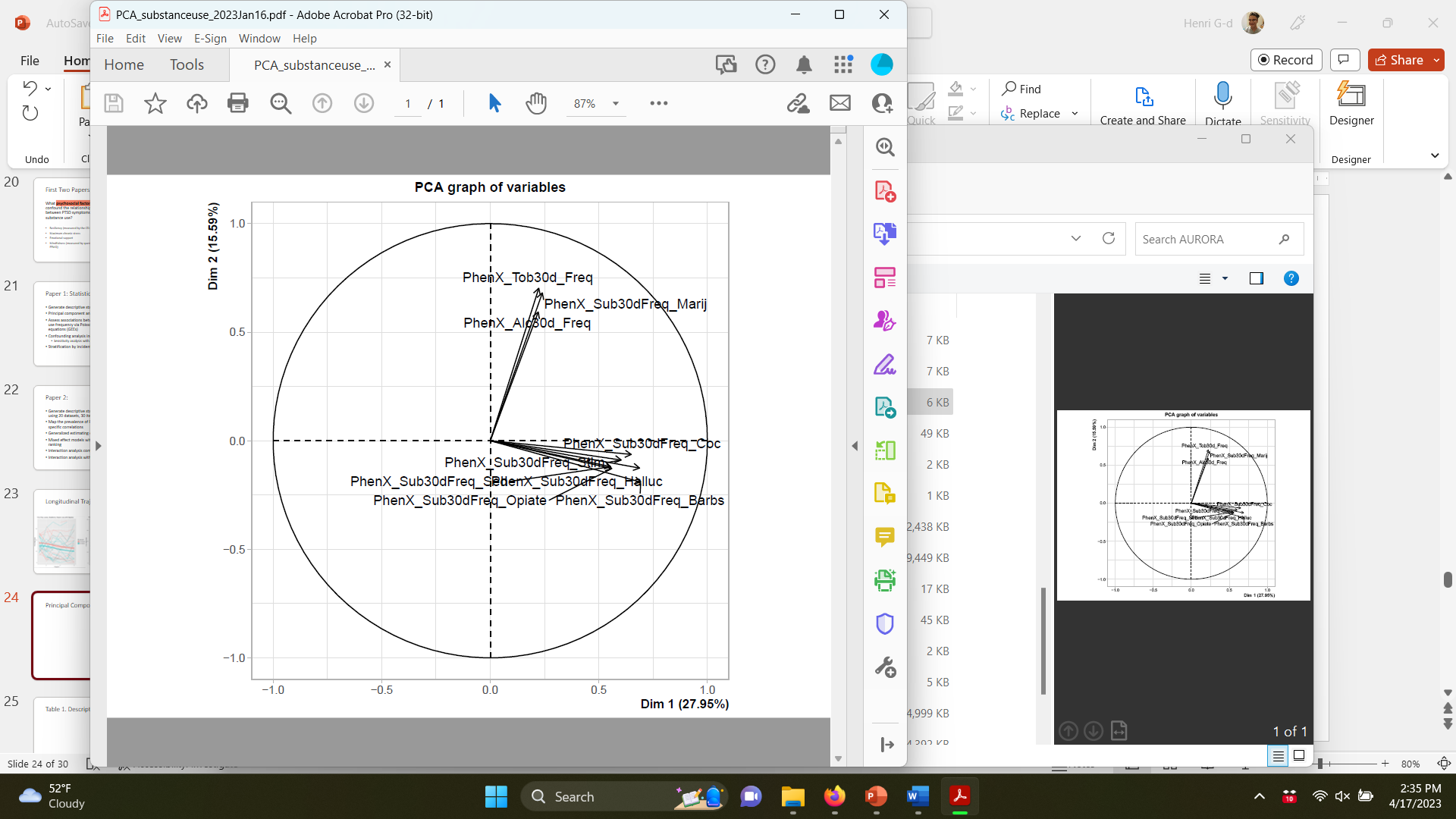
**

**Supplemental Figure 2. Correlogram of past 30 day substance use frequency for tobacco, alcohol, cannabis, cocaine, opiates, stimulants, sedatives, hallucinogens, and barbiturates.**

**
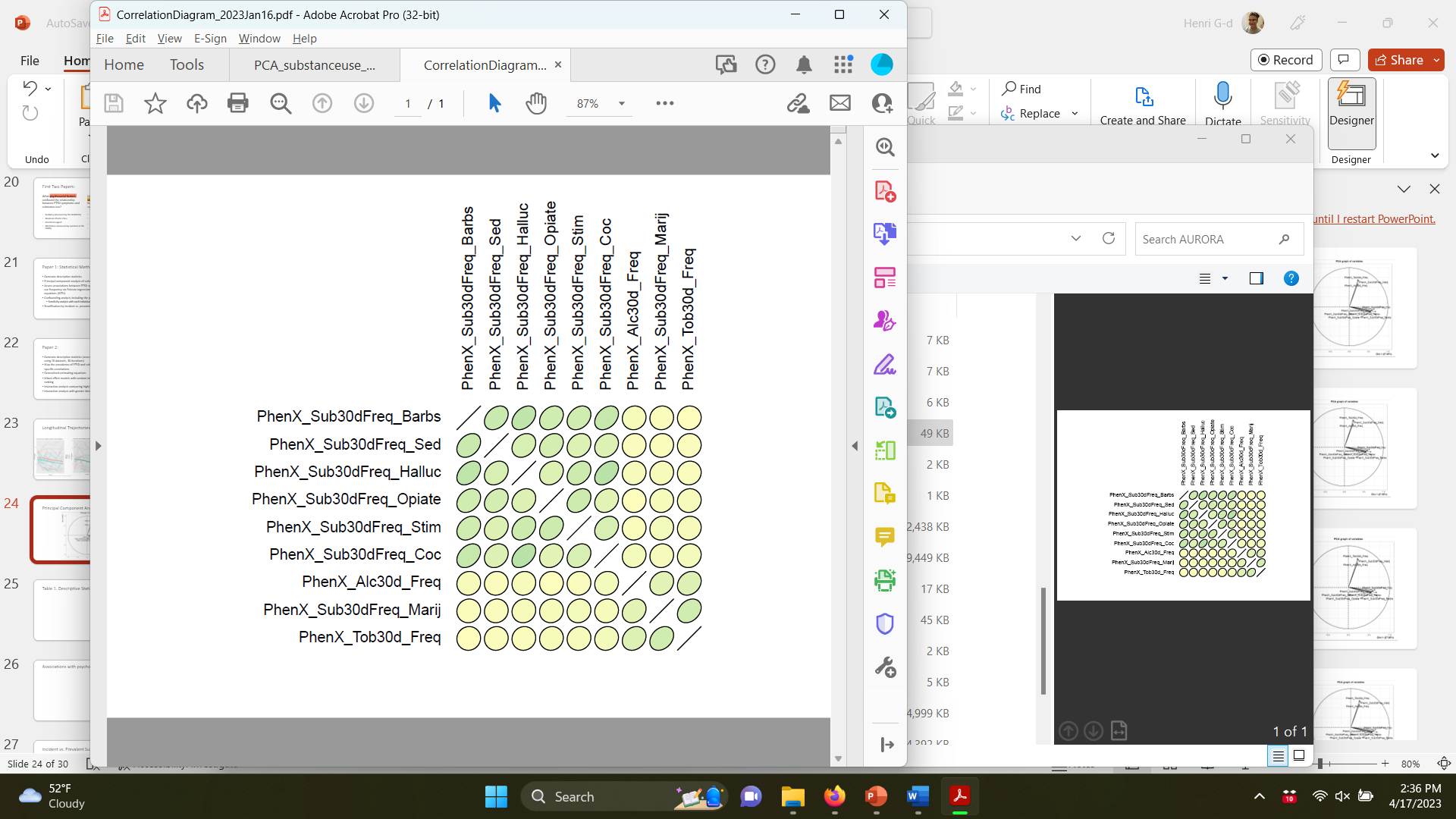
**

**Supplemental Table 1**. Specific class of index traumatic event in AURORA sample.

|  | No likely PTSD  N (%) | Likely PTSD  N (%) |
| --- | --- | --- |
| Motor vehicle collision |  |  |
| Occupant of motor vehicle on a public road | 710 (71.7%) | 596 (63.1%) |
| Rider on a motor vehicle (motorcycle, ATV) on a public road | 31 (3.1%) | 30 (3.2%) |
| Occupant of a motor vehicle off road | 2 (0.2%) | 2 (0.2%) |
| Rider on a motor vehicle (motorcycle, ATV) off road | 3 (0.3%) | 5 (0.5%) |
| Non-occupant hit by a motor vehicle on a public road | 60 (6.1%) | 36 (0.1%) |
| Non-occupant hit by a motor vehicle off road | 3 (0.3%) | 5 (0.5%) |
| Other motor vehicle collision | 0 (0%) | 1 (0.1%) |
| Physical assault |  |  |
| Fight or brawl (participant intentionally started or joined) | 12 (1.2%) | 23 (2.4%) |
| Attacked or jumped (participant was targeted and did not voluntarily participate; includes being struck by thrown object or pushed) | 52 (5.3%) | 118 (12.5%) |
| Other physical assault | 3 (0.3%) | 9 (10.0%) |
| Fall >10 feet |  |  |
| During sport or recreational activity | 0 (0%) | 3 (0.3%) |
| Not during sport or recreational activity | 16 (1.6%) | 13 (1.4%) |
| Mass event |  |  |
| Accidental | 5 (0.5%) | 2 (0.2%) |
| Targeted/involuntary | 0 (0%) | 0 (0%) |
| Fall <10 feet |  |  |
| During sport or recreational activity | 5 (0.5%) | 7 (0.7%) |
| Not during sport or recreational activity | 50 (5.1%) | 56 (5.9%) |
| Poisoning |  |  |
| Accidental | 1 (0.1%) | 0 (0%) |
| Targeted/involuntary | 0 (0%) | 0 (0%) |
| Burn |  |  |
| Accidental | 3 (0.3%) | 7 (0.7%) |
| Targeted/involuntary | 0 (0%) | 0 (0%) |
| Other |  |  |
| Accidental | 30 (3.0%) | 27 (2.9%) |
| Targeted/involuntary | 4 (0.4%) | 5 (0.5%) |

Note: Sexual assault is included in physical assault, under “attacked.”

**Supplemental Table 2**. Generalized estimating equations using Poisson model of post-traumatic stress symptoms and tobacco smoking frequency, controlling for sociodemographic factors using three timepoints.

|  | Tobacco Frequency^2^ | | Tobacco Quantity^3^ | | | Alcohol Frequency | | | Alcohol Quantity^3^ | | | Cannabis | | |
| --- | --- | --- | --- | --- | --- | --- | --- | --- | --- | --- | --- | --- | --- | --- |
|  | Incidence Rate  (95% CI) | P-Value | Incidence Rate  (95% CI) | P-Value | Incidence Rate  (95% CI) | | P-Value | Incidence Rate  (95% CI) | | P-Value | Incidence Rate  (95% CI) | | P-Value |  |
| PTSD Symptoms^1^ | 1.004 (1.001, 1.01) | 0.02 | 1.01 (1.002, 1.01) | 0.004 | 1.002 (1, 1.004) | | 0.12 | 1.004 (1.001, 1.006) | | 0.003 | 1.003 (1, 1.01) | | 0.04 |  |
| Time | 0.96 (0.92, 1.01) | 0.14 | 0.99 (0.92, 1.06) | 0.76 | 1.01 (0.97, 1.05) | | 0.71 | 0.97 (0.93, 1.01) | | 0.16 | 0.98 (0.93, 1.04) | | 0.55 |  |
| Marital status |  |  |  |  |  | |  |  | |  |  | |  |  |
| Married (Ref) |  |  |  |  |  | |  |  | |  |  | |  |  |
| Never married | 1.11 (0.93, 1.33) | 0.26 | 1.10 (0.86, 1.41) | 0.44 | 1.13 (0.98, 1.29) | | 0.10 | 1.17 (1.02, 1.34) | | 0.03 | 1.4 (1.12, 1.73) | | 0.003 |  |
| Divorced | 1.27 (1.05, 1.55) | 0.02 | 1.51 (1.15, 1.97) | 0.003 | 0.98 (0.83, 1.16) | | 0.81 | 1.1 (0.94, 1.29) | | 0.25 | 1.32 (1.02, 1.71) | | 0.04 |  |
| Widowed/Other | 1.06 (0.71, 1.57) | 0.79 | 0.98 (0.59, 1.65) | 0.94 | 1.24 (0.88, 1.75) | | 0.21 | 1.12 (0.8, 1.58) | | 0.51 | 1.36 (0.84, 2.21) | | 0.21 |  |
| Gender |  |  |  |  |  | |  |  | |  |  | |  |  |
| Cisgender female (Ref) |  |  |  |  |  | |  |  | |  |  | |  |  |
| Cisgender male | 1.36 (1.21, 1.52) | <0.001 | 1.45 (1.23, 1.69) | <0.001 | 1.15 (1.04, 1.26) | | 0.004 | 1.24 (1.13, 1.35) | | <0.001 | 1.31 (1.14, 1.49) | | <0.001 |  |
| Transgender | 1.61 (0.53, 4.92) | 0.4 | 2.38 (0.5, 11.46) | 0.28 | 0.89 (0.38, 2.1) | | 0.79 | 0.8 (0.41, 1.55) | | 0.5 | 1.94 (0.66, 5.73) | | 0.23 |  |
| Race/ethnicity |  |  |  |  |  | |  |  | |  |  | |  |  |
| Hispanic (Ref) |  |  |  |  |  | |  |  | |  |  | |  |  |
| Non- Hispanic Black | 0.96 (0.37, 2.49) | 0.93 | 0.78 (0.25, 2.44) | 0.67 | 0.77 (0.35, 1.72) | | 0.53 | 0.89 (0.53, 1.5) | | 0.67 | 0.65 (0.3, 1.4) | | 0.27 |  |
| Non-Hispanic Other | 0.85 (0.31, 2.33) | 0.75 | 0.66 (0.19, 2.3) | 0.52 | 0.67 (0.29, 1.54) | | 0.34 | 0.78 (0.45, 1.38) | | 0.40 | 0.55 (0.24, 1.28) | | 0.17 |  |
| Non- Hispanic White | 1.21 (0.46, 3.16) | 0.70 | 1.14 (0.36, 3.57) | 0.83 | 0.87 (0.39, 1.94) | | 0.73 | 0.92 (0.55, 1.54) | | 0.75 | 0.68 (0.32, 1.47) | | 0.33 |  |
| Age | 0.999 (0.99, 1.004) | 0.58 | 1.004 (0.997, 1.01) | 0.25 | 1.00 (0.995, 1.004) | | 0.83 | 0.99 (0.99, 1) | | 0.001 | 0.98 (0.98, 0.99) | | <0.001 |  |
| Income |  |  |  |  |  | |  |  | |  |  | |  |  |
| >$35k and <=$75k (Ref) |  |  |  |  |  | |  |  | |  |  | |  |  |
| <=$35k | 1.34 (1.14, 1.57) | <0.001 | 1.41 (1.13, 1.77) | 0.003 | 0.89 (0.77, 1.01) | | 0.08 | 0.95 (0.85, 1.07) | | 0.4 | 1.14 (0.94, 1.38) | | 0.18 |  |
| >$75k | 0.67 (0.51, 0.86) | 0.002 | 0.5 (0.32, 0.76) | 0.001 | 1.29 (1.08, 1.53) | | 0.004 | 1.13 (0.98, 1.3) | | 0.11 | 0.79 (0.59, 1.05) | | 0.11 |  |
| Did not report | 1.15 (0.93, 1.44) | 0.20 | 1.24 (0.92, 1.65) | 0.15 | 1.06 (0.9, 1.24) | | 0.49 | 1.001 (0.83, 1.2) | | 0.99 | 1.08 (0.86, 1.37) | | 0.50 |  |

^1^PTSD symptoms were assessed via the PTSD Checklist for the DSM – 5 (PCL-5) given at timepoints week 8, month 3, and month 6 following an index traumatic event.

^2^Substance use variables were recorded as past-month use, given at timepoints month 3, month 6, and month 12 and lagged one timepoint ahead of the PTSD symptom assessments in order to maintain a prospective relationship between PTSD symptoms and substance use.

^3^Quantity was defined as average amount used when a person was typically using. For tobacco, this was equivalent to average amount of cigarettes consumed, and for alcohol this was equivalent to average number of drinks consumed.

**Supplemental Table 3.** List of 128 variables considered as potential effect modifiers of the relationship between PTSD and substance use outcomes.

| Variable Name | Domain | Variable Description |
| --- | --- | --- |
| ED_Event_BroadClass | Traumatic event type | Major event indicator |
| ED_Event_SpecificClass | Traumatic event type | Specific event indicator |
| ED_ChiefPain_MuscSkel | Chief complaint at emergency department visit | Musculoskeletal Pain |
| ED_ChiefPain_Neck | Chief complaint at emergency department visit | Neck pain |
| ED_ChiefPain_Back | Chief complaint at emergency department visit | Back pain |
| ED_ChiefPain_Headache | Chief complaint at emergency department visit | Headache |
| ED_ChiefPain_Lac | Chief complaint at emergency department visit | Lacerration |
| ED_ChiefPain_HeadInj | Chief complaint at emergency department visit | Head injury |
| ED_ChiefPain_Arm | Chief complaint at emergency department visit | Arm pain |
| ED_ChiefPain_Leg | Chief complaint at emergency department visit | Leg pain |
| ED_ChiefPain_Abdomil | Chief complaint at emergency department visit | Abdominal pain |
| ED_ChiefPain_Pelvic | Chief complaint at emergency department visit | Pelvic pain |
| ED_ChiefPain_Chest | Chief complaint at emergency department visit | Chest pain |
| ED_ChiefPain_Breast | Chief complaint at emergency department visit | Breast pain |
| WK2_CTQSF_EmoAbu_RS | Childhood Trauma Questionnaire – Short Form | Childhood emotional abuse (raw score) |
| WK2_CTQSF_PhyAbu_RS | Childhood Trauma Questionnaire – Short Form | Childhood physical abuse (raw score) |
| WK2_CTQSF_SexAbu_RS | Childhood Trauma Questionnaire – Short Form | Childhood sexual abuse (raw score) |
| WK2_CTQSF_EmoNeg_RS | Childhood Trauma Questionnaire – Short Form | Childhood emotional neglect (raw score) |
| WK2_CTQSF_PhyNeg_RS | Childhood Trauma Questionnaire – Short Form | Childhood physical neglect (raw score) |
| WK2_CTQSF_Total_RS | Childhood Trauma Questionnaire – Short Form | Total childhood abuse and neglect (raw score) |
| ED_BriefPCL_RS | Brief PTSD Check List | Brief PCL raw score |
| ED_BriefPCL | Brief PTSD Check List | Brief PCL binary indicator of likely diagnosis |
| WK2_BriefPCL_RS | Brief PTSD Check List | Brief PCL raw score |
| WK2_BriefPCL | Brief PTSD Check List | Brief PCL binary indicator of likely diagnosis |
| ED_DisturbingMemories | Specific PTSD items from PTSD Checklist for the DSM-5 | Disturbing memories (5 pt scale) |
| WK2_DisturbingMemories | Specific PTSD items from PTSD Checklist for the DSM-5 | Disturbing memories (5 pt scale) |
| ED_FeelingUpset | Specific PTSD items from PTSD Checklist for the DSM-5 | Feeling upset (5 pt scale) |
| WK2_FeelingUpset | Specific PTSD items from PTSD Checklist for the DSM-5 | Feeling upset (5 pt scale) |
| ED_FeelingCutOff | Specific PTSD items from PTSD Checklist for the DSM-5 | Feeling cut-off (5 pt scale) |
| WK2_FeelingCutOff | Specific PTSD items from PTSD Checklist for the DSM-5 | Feeling cut-off (5 pt scale) |
| ED_FeelingIrritable | Specific PTSD items from PTSD Checklist for the DSM-5 | Feeling irritable (5 pt scale) |
| WK2_FeelingIrritable | Specific PTSD items from PTSD Checklist for the DSM-5 | Feeling irritable (5 pt scale) |
| ED_BadDreams | Specific PTSD items from PTSD Checklist for the DSM-5 | Having bad dreams (5 pt scale) |
| WK2_BadDreams | Specific PTSD items from PTSD Checklist for the DSM-5 | Having bad dreams (5 pt scale) |
| ED_RelivingEvent | Specific PTSD items from PTSD Checklist for the DSM-5 | Reliving the event (5 pt scale) |
| WK2_RelivingEvent | Specific PTSD items from PTSD Checklist for the DSM-5 | Reliving the event (5 pt scale) |
| ED_StrongPhysicalReactions | Specific PTSD items from PTSD Checklist for the DSM-5 | Strong physical reactivity (5 pt scale) |
| WK2_StrongPhysicalReactions | Specific PTSD items from PTSD Checklist for the DSM-5 | Strong physical reactivitiy (5 pt scale) |
| ED_TroubleRemember | Specific PTSD items from PTSD Checklist for the DSM-5 | Trouble remembering event (5 pt scale) |
| WK2_TroubleRemember | Specific PTSD items from PTSD Checklist for the DSM-5 | Trouble remembering event (5 pt scale) |
| ED_BlamingSelf | Specific PTSD items from PTSD Checklist for the DSM-5 | Blaming yourself for what happened (5 pt scale) |
| WK2_BlamingSelf | Specific PTSD items from PTSD Checklist for the DSM-5 | Blaming yourself for what happened (5 pt scale) |
| ED_FeelingFear | Specific PTSD items from PTSD Checklist for the DSM-5 | Feeling fearful or horror (5 pt scale) |
| WK2_FeelingFear | Specific PTSD items from PTSD Checklist for the DSM-5 | Feeling fearful or horror (5 pt scale) |
| ED_LossOfInterest | Specific PTSD items from PTSD Checklist for the DSM-5 | Lost interest in activities you used to enjoy (5 pt scale) |
| WK2_LossOfInterest | Specific PTSD items from PTSD Checklist for the DSM-5 | Lost interest in activity you used to enjoy (5 pt scale) |
| ED_LackPositiveEmotions | Specific PTSD items from PTSD Checklist for the DSM-5 | Trouble experiencing positive emotions (5 pt scale) |
| WK2_LackPositiveEmotions | Specific PTSD items from PTSD Checklist for the DSM-5 | Trouble experiencing positive emotions (5 pt scale) |
| ED_TakingRisks | Specific PTSD items from PTSD Checklist for the DSM-5 | Taking too many risks (5 pt scale) |
| WK2_TakingRisks | Specific PTSD items from PTSD Checklist for the DSM-5 | Taking too many risks (5 pt scale) |
| ED_Superalert | Specific PTSD items from PTSD Checklist for the DSM-5 | Being superalert or watchful (5 pt scale) |
| WK2_Superalert | Specific PTSD items from PTSD Checklist for the DSM-5 | Being superalert or watchful (5 pt scale) |
| ED_FeelingJumpy | Specific PTSD items from PTSD Checklist for the DSM-5 | Feeling jumpy or easily startled (5 pt scale) |
| WK2_FeelingJumpy | Specific PTSD items from PTSD Checklist for the DSM-5 | Feeling jumpy or easily startled (5 pt scale) |
| ED_SleepProblems | Specific PTSD items from PTSD Checklist for the DSM-5 | Trouble falling or staying asleep (5 pt sclae) |
| WK2_SleepProblems | Specific PTSD items from PTSD Checklist for the DSM-5 | Trouble falling or staying asleep (5 pt scale) |
| Wk2_Polytrauma | Polytrauma | Defined as positively endorsing 2 or more: PTSD symptoms, somatic symptoms, and pain symptoms |
| WK2_PROM_EmoSup4a_RS | PROMIS emotional support metric | Emotional support (raw score) |
| WK2_AffInt_Ppl_Num | Supportive and negative social interaction scale | Number of social interactions in the past month (raw score) |
| WK2_AffInt_Ppl_Num_pre_std | Supportive and negative social interaction scale | Number of social interactions in the past month (standardized) |
| WK2_AffInt_Ppl_Freq_RS | Supportive and negative social interaction scale | Frequency of social interacctions (how often) |
| WK2_AffInt_Grp_Freq_RS | Supportive and negative social interaction scale | Frequency of social interactions with affiliated social or religious groups |
| WK2_SIS_NetPos_RS | Supportive and negative social interaction scale | Network positive interactions for social support with a serious issue (raw score) |
| WK2_SIS_NetPos_Num | Supportive and negative social interaction scale | Number of network positive relationships you can rely on (number) |
| WK2_SIS_NetPos_Num_pre_std | Supportive and negative social interaction scale | Number of network positive relationships you can rely on (standardized) |
| WK2_SIS_NetPos_Conf | Supportive and negative social interaction scale | Number of network positive relationships you can confide in (number) |
| WK2_SIS_NetPos_Conf_pre_std | Supportive and negative social interaction scale | Number of network positive relationships you can confide in (standardized) |
| WK2_CDRISC10_RS | Connor-Davidson Resiliency Scale – 10 | Ability to cope with adversity (10 item scale) |
| ED_Stress_MAX | Chronic Stress scale (10 point) | Maximum stress severity in past 30 days |
| WK2_Stress_MAX | Chronic Stress scale (10 point) | Maximum stress severity in past 30 days |
| WK2_FFMQ_RS | Five-Facet Mindfulness Questionnaire | Mindfulness related to observing, describing, acting with awareness. Non-judgment of experience, non-reacitivity to inner experience |
| ED_PSQI_SlpDur_RS | Pittsburgh Sleep Quality Index | Sleep hours/night |
| WK2_PSQI_SlpDur_RS | Pittsburgh Sleep Quality Index | Sleep hours/night |
| ED_PSQI_SlpDur_Gen_RS | Pittsburgh Sleep Quality Index | Sleep hours/night in general |
| ED_PSQI_SlpDur_Gen | Pittsburgh Sleep Quality Index | Sleep hours/night in general |
| ED_PSQI_SlpDur | Pittsburgh Sleep Quality Index | Sleep quality per night |
| WK2_PSQI_SlpDur | Pittsburgh Sleep Quality Index | Sleep quality per night |
| ED_ISI_RS | Insomnia Severity | Insomnia severity raw score |
| WK2_ISI_RS | Insomnia Severity | Insomnia severity raw score |
| ED_ISI | Insomnia Severity | Non-significant, subthreshold, clinical moderate, or clinical severe insomnia |
| WK2_ISI | Insomnia Severity | Non-significant, subthreshold, clinical moderate, or clinical severe insomnia |
| ED_RaceEthCode | Race/ethnicity | Hispanic, non-Hispanic Black, non-Hispanic White, Asian American/Pacific Islander, American Indian/Native American, other |
| CensusDivision | Census division |  |
| CensusRegion | Census region |  |
| ADI_tRank | Area deprivation index | Total national rank percentile |
| ADI_StateRank | Area deprivation index | Within-state decile |
| ED_Age | Individual sociodemographic factor | Age at enrollment |
| ED_GenderBirthCert | Individual sociodemographic factor | Male, female |
| ED_GenderNow | Individual sociodemographic factor | Male, female, transgender, do not identify |
| ED_Marital | Individual sociodemographic factor | Never married, divorced, separated, annulled, widowed, married |
| ED_HighestGrade | Individual sociodemographic factor | Numeric highest grade achieved, some college no degree, associate degree, bachelor’s degree, master’s degree, professional school degree, doctoral degree |
| WK2_EmploymentCode | Individual sociodemographic factor | Current employment: Currently work, laid off, on leave, other on leave, retired, homemaker, student, unemployed, disabled, other work |
| WK2_IncomeCode | Individual sociodemographic factor | Household income:  <$19k, $19001 to $35k, $35001 to $50k, $50001 to $75k, $75001 to $100k, >=$100k |
| ED_SF12_PCS | Short Form Health Survey (12-item) | Measure of general health, 2 summary scales and 4 health concepts in each scale |
| WK2_SF12_PCS | Short Form Health Survey (12-item) | Measure of general health, 2 summary scales and 4 health concepts in each scale |
| ED_PROM_Dep8b_RS | PROMIS Depression scale | Shortform depression score (raw score) |
| ED_PROM_Dep8b_T | PROMIS Depression scale | Shortform depression score (T score) |
| ED_PROM_Dep8b_SE | PROMIS Depression scale | Shortform depression score (standard error of T score) |
| ED_PROM_Dep8b | PROMIS Depression scale | Binary indicator of likely depression |
| WK2_PROM_Dep8b_RS | PROMIS Depression scale | Shortform depression score (raw score) |
| WK2_PROM_Dep8b_T | PROMIS Depression scale | Shortform depression score (T score) |
| WK2_PROM_Dep8b_SE | PROMIS Depression scale | Shortform depression score (standard error of T score) |
| WK2_PROM_Dep8b | PROMIS Depression scale | Binary indicator of likely depression |
| ED_PROM_AnxBank_RS | PROMIS Anxiety Bank Items | Anxiety sensitivity index (raw score) |
| WK2_PROM_AnxBank_RS | PROMIS Anxiety Bank Items | Anxiety sensitivity index (raw score) |
| ED_SCRN_GAD_RS | PROMIS Generalized Anxiety Disorder Screener | Generalized anxiety screener score (raw score) |
| WK2_SCRN_GAD_RS | PROMIS Generalized Anxiety Disorder Screener | Generalized anxiety screener score (raw score) |
| state | Participant state | State of residence at enrolment |
| ED_Event_BroadClass_fct | Sociodemographic factor (recode) |  |
| ED_Marital_fct | Sociodemographic factor (recode) |  |
| ED_GenderNow_fct | Sociodemographic factor (recode) |  |
| edu | Sociodemographic factor (recode) |  |
| marital | Sociodemographic factor (recode) |  |
| gender | Sociodemographic factor (recode) |  |
| income | Sociodemographic factor (recode) |  |
| lt_sub_age | Lifetime substance use | Age of initation of lifetime substance use |
| lt_sub_yrs | Lifetime substance use | Years of lifetime substance use |
| lt_sub_maxfreq | Lifetime substance use | Maximum frequency of substance use during period highest lifetime use |
| time | Time period | Retrospective recall of 30 days before ED, ED baseline, week 2, week 8, month 3, month 6, month 12 (categorical) |
| time2 | Time period (recode) | Time variable recoded as ordinal |
| LT_youtrauma | Life Events Checklist | Experienced traumatic event directly |
| LT_witnesstrauma | Life Events Checklist | Witnessed traumatic event happen to someone else |
| LT_jobtrauma | Life Events Checklist | Exposed to traumatic events as part of your job |
| LT_sotrauma | Life Events Checklist | Something traumatic happened to someone close to you |
| lt_ciguse_wrst | Lifetime substance use | Lifetime worst cigarette use during year of highest use |
| trauma.type | Traumatic event type (recode) |  |
| employ | Sociodemographic factor (recode) | Recoded employment |
| adi_cat | Sociodemographic factor (recode) | Recoded area deprivation index category |

**Supplemental Table 4.** Variable cutoffs of stratification tau predictions using 25^th^/75^th^ and 10^th^/90^th^ cutoff of conditional average treatment effects.

| Substance^2^ | Less than 25^th^ | Greater than 75^th^ | Less than 10^th^ | Greater than 90^th^ |
| --- | --- | --- | --- | --- |
|  | ATE^1^ (95% CI) | ATE (95% CI) | ATE (95% CI) | ATE (95% CI) |
| Tobacco | -0.02 (-0.08, 0.05) | 0.08 (0.004, 0.16) | -0.02 (-0.16, 0.11) | 0.17 (0.03, 0.31) |
| Alcohol | -0.03 (-0.04, -0.02) | 0.001 (-0.03, 0.03) | -0.04 (-0.05, -0.02) | -0.003 (-0.02, 0.01) |
| Cannabis | 0.004 (-0.02, 0.02) | 0.01 (-0.005, 0.02) | 0.01 (-0.02, 0.02) | 0.02 (0.002, 0.04) |

^1^ ATE: Average Treatment Effect, indicative of the effect of PTSD on prospective substance use using 3-timepoint lagging and linear model.

^2^Substance use was assessed as frequency of use in the past 30 days.

Tau predictions are the average treatment effect conditioned on all included moderators in the causal forest. Estimates from the causal forest are used to predict the relationship between PTSD and substance use, maximizing the difference in the stratified estimates at each decision node. Aggregating across the trees, we may use the predicted tau similar to a propensity score, where it represents the overall effect of all moderators for the conditional ATE.

**
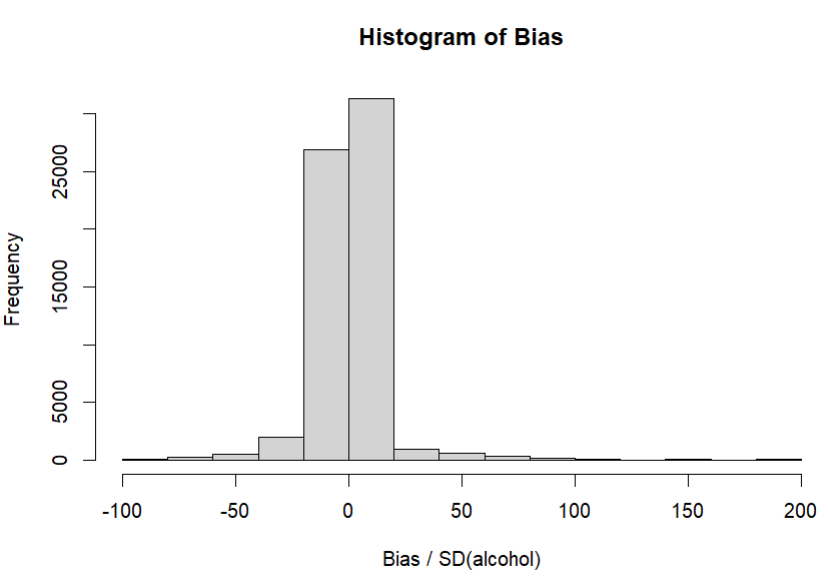

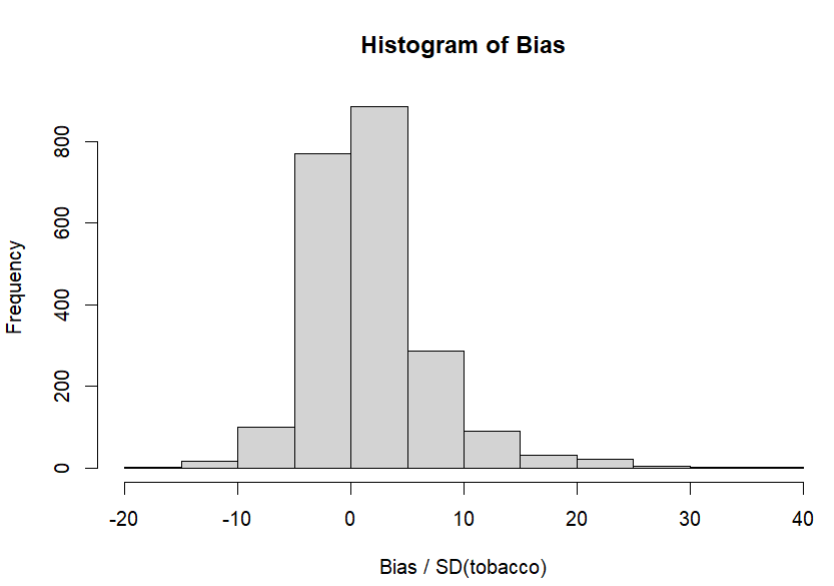
Supplmental Figure 3**. Histogram of bias in causal forest predictions compared to standard deviation of substance use for tobacco, alcohol, and cannabis.


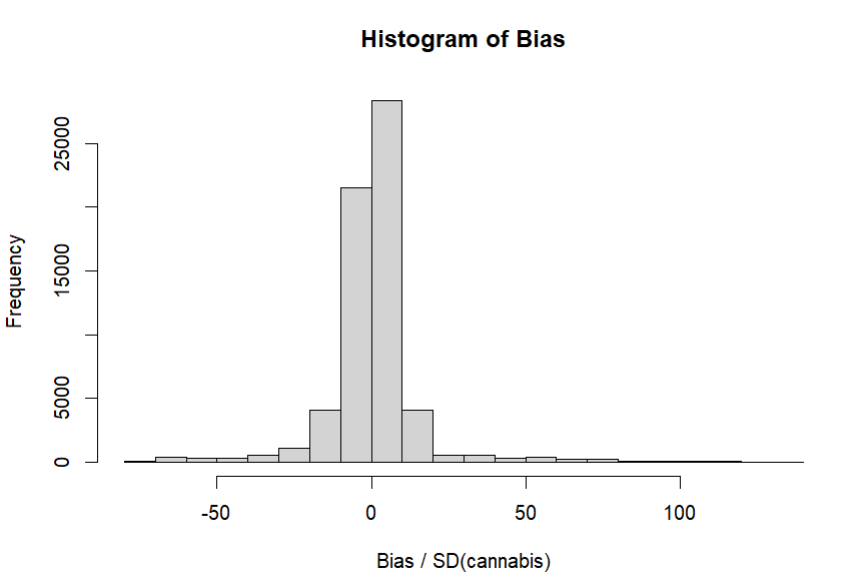


Bias estimates were generated *bias*(*x*)=(*e*(*x*)−*p*)×(*p*(*μ*(0,*x*)−*μ*0)+(1−*p*)(*μ*(1,*x*)−*μ*1), in short, the bias is the difference between the observed average treatment effect and the estimated average treatment effect (tau), calculated proportional to the covariance of the propensity score and the weighted average of conditional expected, as per Athey et al., 2017. Bias estimates centered around 0 suggest reasonable fit.

Frequency estimates reflect across all datasets used in multiple imputation (20 datasets)
